# Nutrient depletion-induced production of tri-acylated glycerophospholipids in *Acinetobacter radioresistens*

**DOI:** 10.1038/s41598-018-25869-9

**Published:** 2018-05-10

**Authors:** Yu Luo, Muhammad Afzal Javed, Harry Deneer, Xialu Chen

**Affiliations:** 1 Department of Biochemistry, College of Medicine, University of Saskatchewan, Saskatoon, Saskatchewan Canada; 2 Department of Microbiology and Immunology, College of Medicine, University of Saskatchewan, Saskatoon, Saskatchewan Canada; 3 Department of Pathology and Laboratory Medicine, College of Medicine, University of Saskatchewan, Saskatoon, Saskatchewan Canada; 4 Molecular Microbiology Laboratory, Division of Clinical Microbiology, Saskatoon Health Region, Saskatoon, Saskatchewan Canada

## Abstract

Bacteria inhabit a vast range of biological niches and have evolved diverse mechanisms to cope with environmental stressors. The genus *Acinetobacter* comprises a complex group of Gram-negative bacteria. Some of these bacteria such as *A. baumannii* are nosocomial pathogens. They are often resistant to multiple antibiotics and are associated with epidemic outbreaks. *A. radioresistens* is generally considered to be a commensal bacterium on human skin or an opportunistic pathogen. Interestingly, this species has exceptional resistance to a range of environmental challenges which contributes to its persistence in clinical environment and on human skin. We studied changes in its lipid composition induced by the onset of stationary phase. This strain produced triglycerides (TG) as well as four common phospholipids: phosphatidylethanolamine (PE), phosphatidylglycerol (PG), cardiolipin (CL) and lysocardiolipin (LCL). It also produced small amounts of acyl-phosphatidylglycerol (APG). As the bacterial growth entered the stationary phase, the lipidome switched from one dominated by PE and PG to another dominated by CL and LCL. Surprisingly, bacteria in the stationary phase produced N-acyl-phosphatidylethanolamine (NAPE) and another rare lipid we tentatively name as 1-phosphatidyl-2-acyl-glycero-3-phosphoethanolamine (PAGPE) based on tandem mass spectrometry. It is possible these tri-acylated lipids play an important role in coping with nutrient depletion.

## Introduction

Bacteria thrive in every corner of the biosphere. They have evolved a diverse array of survival adaptations to cope with environmental stressors such as starvation, desiccation, change in temperature, pH and salinity. As the permeation barrier, the bacterial membrane stands at the forefront to sense and relay signals of environmental change for metabolic adaptation. Noticeable changes in the lipidome have been associated with fatty acid composition which tunes permeability and flexibility of the lipid bilayer^1,2^. For instance, saturated fatty acids are known to increase membrane rigidity, while cis-monounsaturated fatty acids and similarly kinked cyclopropane fatty acids tend to do the exact opposite^1^. Among extremophiles, bacteria are rarely observed to form tetraether monolayer membranes as frequently observed in archaea, which are superior to fatty acyl ester-based bacterial lipid bilayers in surviving extremely high temperature, high salinity and low pH^2^. On the other extreme of pH and temperature, bacteria have a slight adaptive edge over archaea. They are often observed to reduce fatty acids with iso- and anteiso-methyl branches to better cope with low temperature^2^. The polar lipid head groups have also been associated with lipidome adaptation to stressors. Bacteria thriving at extremely high pH tend to produce more cardiolipin (CL) as well as bis-mono-acyl-glycero-phosphate (BMP)^3^. In stationary phase, cardiolipin synthase activity has been observed to increase approximately 10-fold in the Gram-negative model organism *Escherichia coli*, suggesting CL is an essential membrane component to cope with starvation^4^. In Gram-positive *Bacillus subtilis*, the transcriptional activity of cardiolipin synthase has also been observed to increase 2-fold upon entry into stationary phase or under increased salinity^5^. While CL increases, the two predominant lipids phosphatidylglycerol (PG) and phosphatidylethanolamine (PE) of the exponential growth phase decrease as *B. subtilis* enters stationary phase^6^. We expected to observe similar changes in relative abundances of these major polar lipids as *A. radioresistens* enters stationary phase.

*Acinetobacter radioresistens* is a Gram-negative species of remarkable resistance to radiation and antibiotics^7^. It is often a harmless bacterium isolated from soil, water and dry environment including the human skin, but has also recently been recognized as an emerging pathogen of humans^8^. Like the more virulent *A. baumannii*, *A. radioresistens* is often resistant to multiple antibiotics and can cause catheter-related nosocomial bloodstream infection and community-acquired infection in HIV-positive patients^9^. Due to its extreme survival capability to withstand prolonged periods of desiccation^10^, it would be an ideal organism to study for its adaptation to cope with long-term starvation and desiccation. Based on genetic analysis, the *A. radioresistens* clad is placed close to the root of the phylogenetic tree of the genus *Acinectobacter*, and has the smallest genome size^11,12^. Most evolved strategies in *A. radioresistens* are expected to be relevant in more virulent strains such as *A. baumannii* which has an expanded genome.

We have recently employed mass spectrometry in lipid profiling of Gram-positive bacteria *Bacillus subtilis*^13^ and reported the discovery of novel lipids such as N-succinyl-lysyl-PG from *Staphylococcus haemolyticus*^14^. By searching for precursors of *m/z* 140 deprotonated phosphoethanolamine head group fragment in lipids extracted from Gram-negative bacteria *Acinetobacter radioresistens*, we observed a lipid species similar in size to lysocardiolipin (LCL), and tentatively assigned its chemical structure as 1-phosphatidyl-2-acyl-glycero-3-phophoethanolamine (PAGPE). Importantly, PAGPE was only observed in the stationary phase. Since the lipidome of this bacterium was also observed to transition from a PE and PG-dominated state in the exponential growth phase to a CL and LCL-dominated state in the stationary phase, we postulate that PAGPE with a compatible shape to LCL and CL may serve as a better charge modulator than the much smaller PE in the bacterial membrane during stationary phase. We also observed small amounts of two additional tri-acylated phospholipids: acyl-phosphatidylglycerol (APG) and N-acyl-phosphatidylethanolamine (NAPE). Importantly, production of NAPE was also triggered by the onset of stationary phase. The lipid extracts also contained large amounts of triglycerides (TG) which likely serves as an fatty acid reserve. Biosynthesis of these tri-acylated species appear to be an evolved adaptation in *A. radioresistens*.

## Results

### Profiling and tandem mass spectrometry of lipid extract from *A. radioresistens* in exponential growth phase

**Figure 1:**
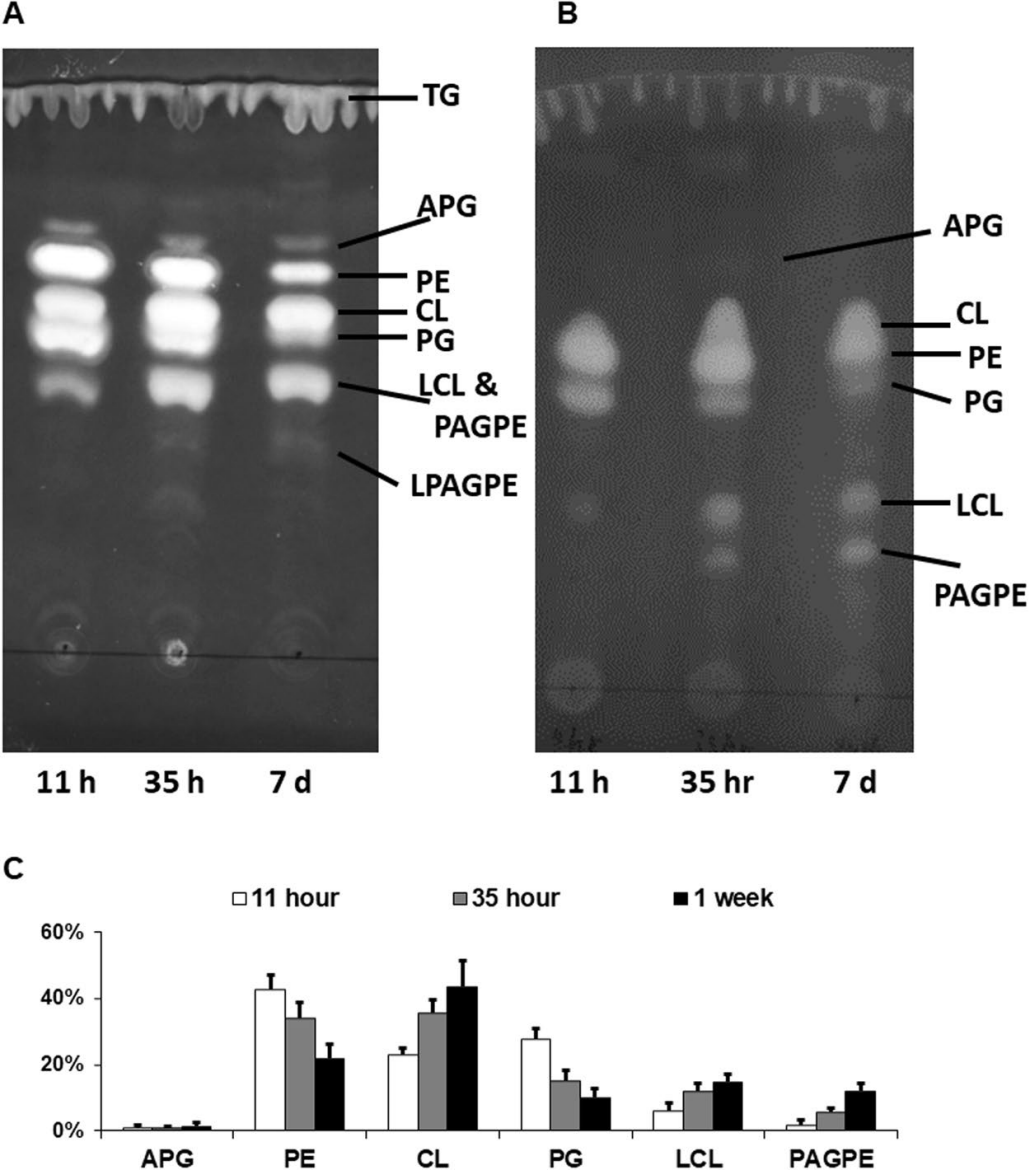
Thin-layer chromatogram and lipid composition of lipids extracted from *A. radioresistens*. TG (triglyceride), PG (phosphatidylglycerol), APG (acyl-phosphatidylglycerol), PAGPE (1-phosphatidyl-2-acyl-glycero-3-phosphoethanolamine, PE (phosphatidylethanolamine), CL (cardiolipin) and LCL (lysocardiolipin) were separated by TLC (thin-layer chromatography). (**A**) Thin-layer chromatogram developed in 75:25:4 chloroform/methanol/water. (**B**) Thin-layer chromatogram developed in 75:25:3:1 chloroform/methanol/water/acetic acid (glacial). The TLC sheets were stained with 0.02% primuline in 80:20 acetone/water. Representative fluorescent bands were analyzed by MS for identification. The lipid compositions are marked on the right. TG was likely diffused along the solvent front. (**C**) Semi-quantitative estimate of lipid composition based on primuline fluorescence.

**Figure 2:**
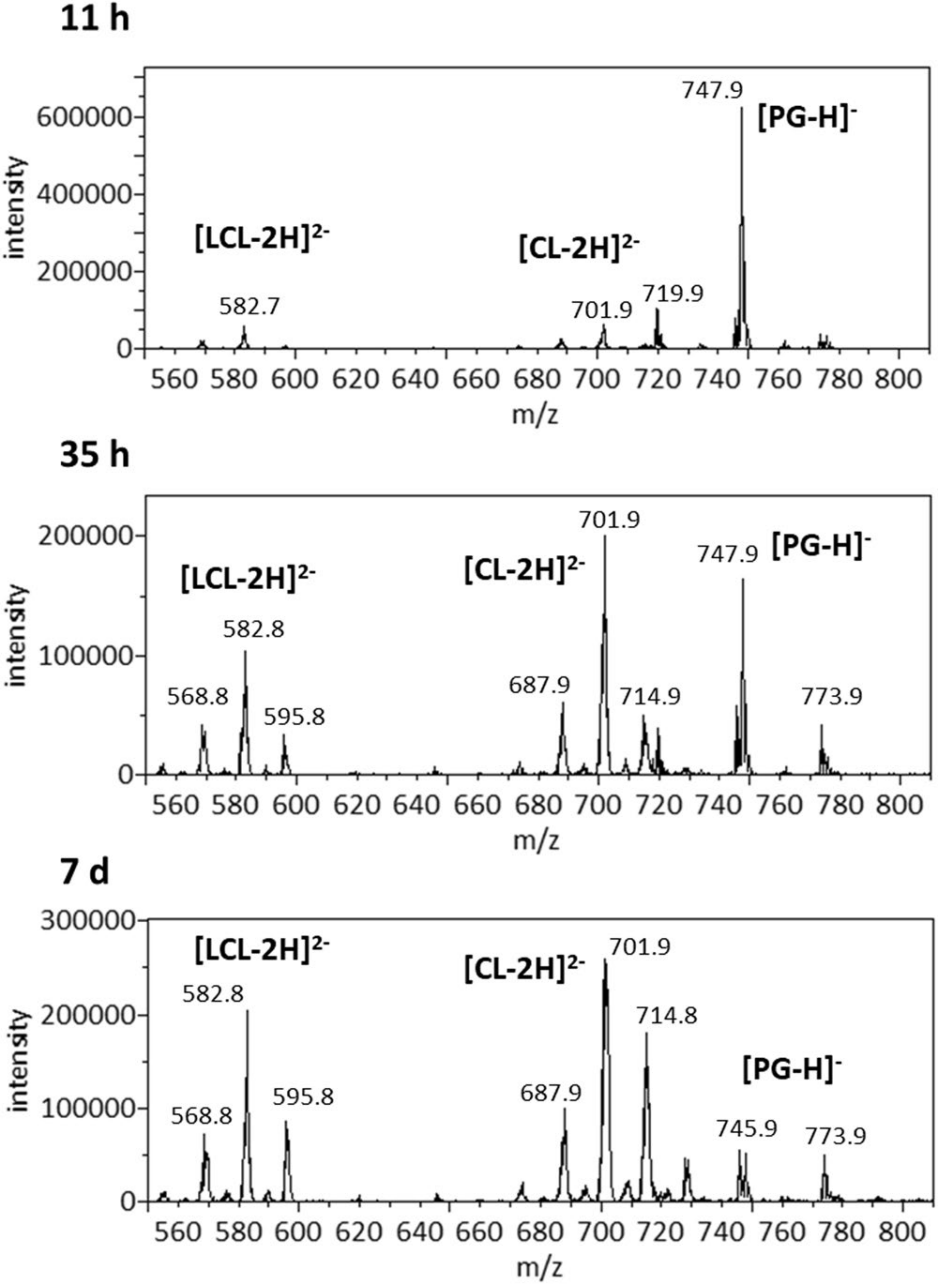
Precursor scans for m/z 153 anionic head group fragment. The most abundant peaks of phosphatidylglycerol (PG) anions (*m/z* 747), CL double anions (*m/z* 700–701) and lysocardiolipin (LCL) double anions (*m/z* 582) are labeled. Top: Cells were grown for 11 h after inoculation. Middle: Cells were grown for 35 h. Bottom: Cells were grown for 7 d.

**Figure 3:**
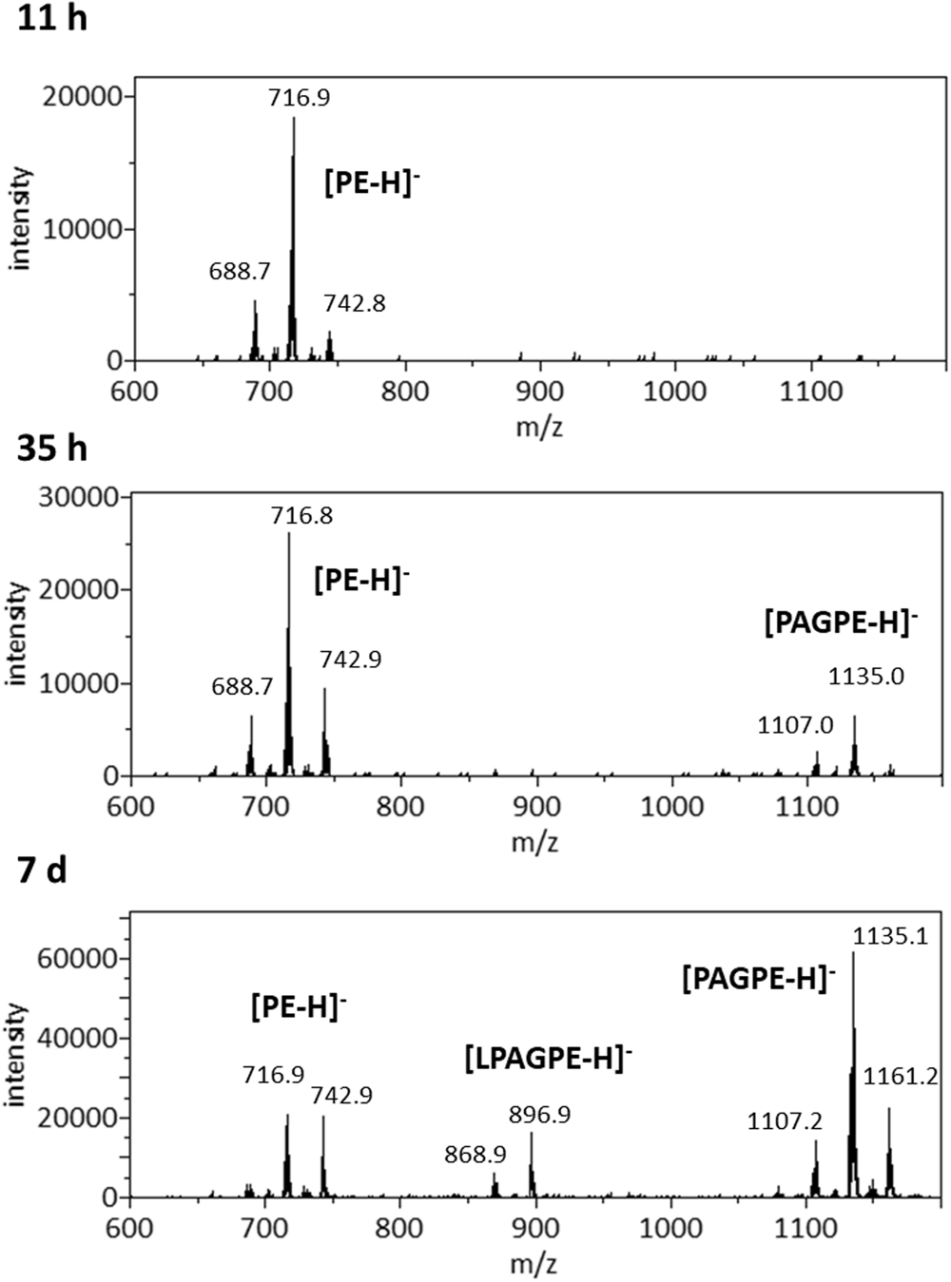
Precursor scans for m/z 140 anionic head group fragment. The most abundant peaks of phosphatidylethanolamine (PE), 1-phosphatidyl-2-acyl-glycero-3-phosphoethanolamine (PAGPE) and lyso-1-phosphatidyl-2-acyl-glycero-3-phosphoethanolamine (LPAGPE) anions are labeled. Top: Cells were grown for 11 h after inoculation. Middle: Cells were grown for 35 h. Bottom: Cells were grown for 7d.

**Figure 4:**
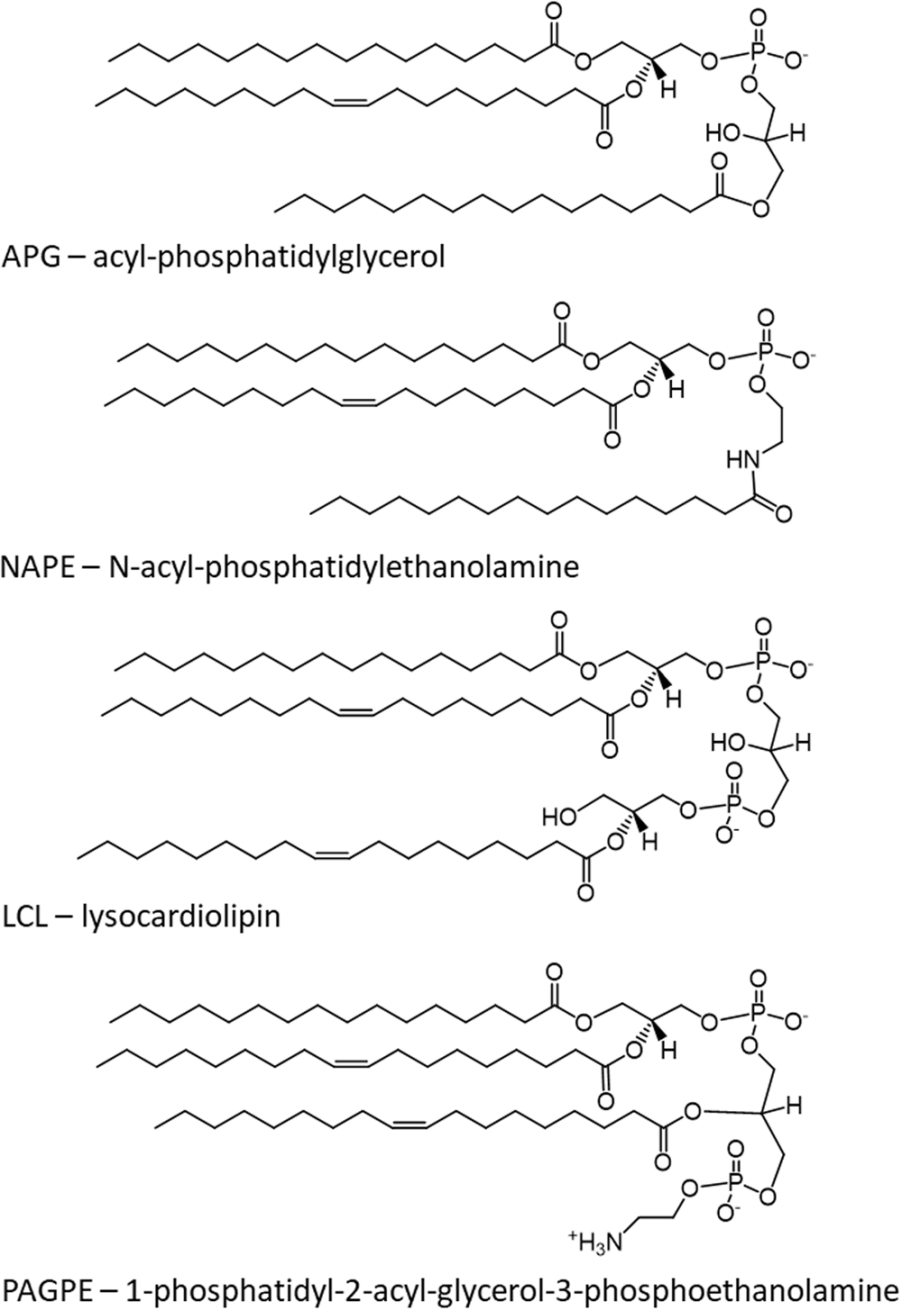
Summary of chemical structures of tri-acylated glycerophospholipids. Chemical structures of acyl-phosphatidylglycerol (APG), N-acyl phosphatidylethanolamine (NAPE), lysocardiolipin (LCL), and 1-phosphatidyl-2-acyl-glycero-3-phosphoethanolamine (PAGPE) are shown. The predominant (16:0–18:1) phosphatidyl group shared by these lipids is shown in these structures. The third predominant fatty acyl group had a composition of either (16:0) or (18:1), as shown in this figure.

We first extracted polar lipids from this *A. radioresistens* species using the Bligh-Dyer method^15^ and carried out lipid profiling by mass spectrometry (MS) and thin-layer chromatography (TLC, Fig. 1). In addition to polar lipids, we also observed an abundance of ammoniated cations of TG in the positive mass spectrum (Supplementary Fig. S1 and Table S1). TG was also partially recovered from silica gels scraped from the solvent front on the TLC sheet (Fig. 1). The MS/MS spectra of these TG ammonium adduct ions matched the dissociation pattern that has been previously published^16^. The negative mass spectrum was dominated by deprotonated PG ions, followed by those of PE, CL and LCL. A precursor scan for the *m/z* 153 cyclo-phosphoglycerol anion (Fig. 2) highlighted the anions of PG, CL and LCL. PE anions were highlighted by a precursor scan for the *m/z* 140 phosphoethanolamine head group anion (Fig. 3). The MS/MS spectra of these four common glycerophospholipids matched their known dissociation features^17^ and enabled us to decipher their fatty acid compositions (Supplementary Figs S2, S3, S5 and S6). These glycerophospholipids were also observed as major primuline-stained fluorescent bands by TLC analysis (Fig. 1). Integrated fluorescence intensity was used to estimate percentage of each type of lipids (Fig. 1C). We were able to carry out a thorough survey of fatty acid compositions by tandem mass spectrometry in the most abundant PG anions (Supplementary Table S2). The predominant fatty acid composition was found to be (16:0) at the sn-1 position and (18:1) at the sn-2 position, which corresponds to the *m/z* 747 PG anion and *m/z* 716 PE anion (Figs 2 and 3). This fatty acid configuration also corresponds to the major CL double anion at *m/z* 701 and the major LCL double anion at *m/z* 582 (Fig. 2). A tri-acylated lipid as a minor fast-migrating species on the TLC sheet (Fig. 1) was also identified as tri-acylated APG by tandem mass spectrometry (Supplementary Fig. S4 and Table S4), which matched the dissociation pattern that has been observed for a chemically synthesized reference compound^18^. Chemical structures of four tri-acylated glycerophospholipids identified in *A. radioresistens* are summarized in Fig. 4.

### Onset of stationary phase-triggered production of tri-acylated phospholipids in *A. radioresistens*

Starvation is a norm rather than exception for most bacteria. It would be interesting to monitor lipidome adaptation of bacteria as nutrients are depleted. We initially analyzed the bacterial lipid extracts at multiple time points between 5 and 36 hours after inoculation with overnight culture, representing early exponential growth phase to early stationary phase. The lipidome composition appeared quite stable during the exponential growth phase, but seemed to include additional types of lipids during the stationary phase.

**Figure 5:**
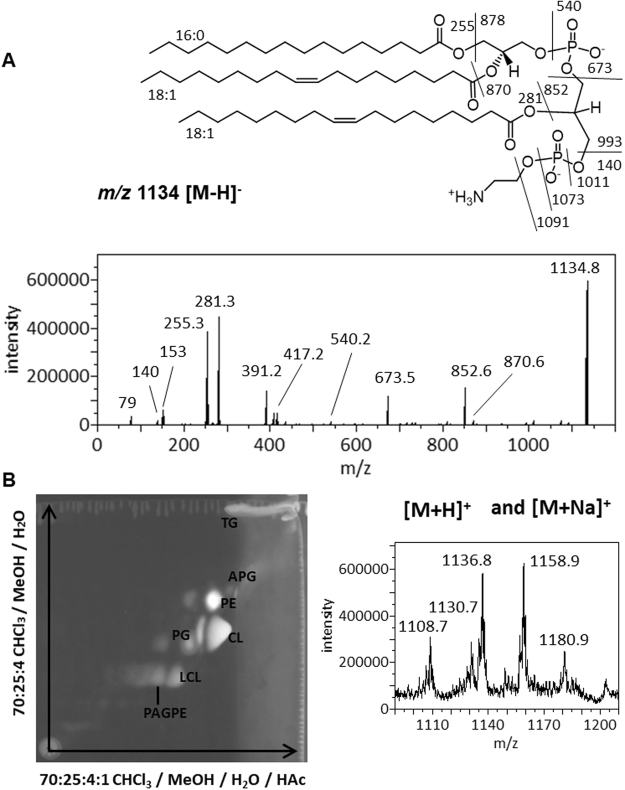
MS/MS spectrum of the m/z 1134 PAGPE anion and 2D-TLC chromatogram. (**A**) Dissociation and MS/MS spectrum of the *m/z* 1134 deprotonated anion. (**B**) The 2-D-TLC sheet was stained with 0.02% primuline. Representative fluorescent spots were analyzed by MS. The types of lipids recovered from silica gels are marked on the chromatogram. Positive MS spectrum of lipids recovered at the PAGPE spot is shown.

An emerging lipid species during the stationary phase was noticed first by a precursor scan for PE and similar lipids which produce a *m/z* 140 anionic head group fragment of phosphoethanolamine (Fig. 3), implying a terminal phosphoethanolamine in its structure. We further acquired and manually interpreted the MS/MS spectra of this species in negative mode (Fig. 5A and Supplementary Fig. S7A). The predominant anion of this type was observed at *m/z* 1134. It was also revealed as minor peaks in precursor scans for *m/z* 79 phosphite anion, *m/z* 97 phosphate anion, and *m/z* 153 cyclic phosphoglycerol anion. Collectively, these precursor scans suggest that it is likely a glycerophospholipid, which can be identified by MS/MS analysis based on known patterns of dissociation at either ester or phosphoester bonds^17^. Two signature fragments of PE head group at *m/z* 140 and 196 were observed, corresponding to phosphoethanolamine and cyclic glycero-phosphoethanolamine anions, respectively. There were two dominant fatty acid anions at *m/z* 255 and 281, respectively. However, the intensities of these two fatty acid anions were too similar for assigning them to the sn-1 and sn-2 positions. Cyclic lyso-phosphatidic acid (lyso-PA) anions with (16:0) and (18:1) fatty acyl would match the fragments observed at *m/z* 391 and 417, respectively. Neutral loss of (18:1) FA or as ketene would produce fragments at 852 and 870 m/z (unlabelled in Fig. 5A), respectively. Neutral loss of the (16:0) FA would produce the much weaker *m/z* 878 anion (unlabelled in Fig. 5A). Importantly, the *m/z* 673 ion would match a phosphatidic acid (PA) anion with (34:1) fatty acyl composition, which possibly corresponds to the most common (16:0–18:1) composition observed among PG, PE and CL. Neutral loss of a DG (diglyceride) of this composition (594 amu) would produce the observed *m/z* 540 ion with a (18:1) fatty acyl group. Neutral loss of ethanolamine (61 amu) or cyclo-ethylamine (43 amu) would produce the pair of *m/z* 1073 and 1091 ions, while loss of linear or cyclic phosphoethanolamine (141 or 123 amu) would produce the pair of *m/z* 993 and 1011 ions. Observation of these four anionic fragments strongly suggest that the ethanolamine moiety is attached at the terminus, which would require that the third fatty acyl be attached to the central glycerol. This tandem MS spectrum, as well as the fact that phosphodiester bonds are biosynthetically formed only with the terminal hydroxyls in glycerol, collectively suggest a tri-acylated structure we tentatively name as 1-phosphatidyl-2-acyl-glycero-3-phospho-ethanolamine (PAGPE), as shown in Fig. 4. In late stationary phase, a possible lysoform of PAGPE, LPAGPE, was observed as a *m/z* 896 anion by MS analysis (Figs 1 and 3). Manual interpretation of the negative MS/MS spectrum of the *m/z* 896 species (Supplementary Fig. S8) appeared to further support the assignment of the polar head group of PAGPE based on the negative MS/MS spectrum of the *m/z* 1134 species.

The putative head group of PAGPE resembles the overall architecture of CL with two phosphodiester bonds, but with one terminal glycerol substituted for ethanolamine. In this structure, the three hydroxyls of the two glycerol moieties, which are not engaged in phosphodiester bonds, form fatty acyl esters (Fig. 4).

By comparing the negative MS/MS spectrum of the *m/z* 1165 LCL single anion (Supplementary Fig. S6B) and the *m/z* 1134 PAGPE anion (Fig. 5A), which have the same fatty acyl composition as well as an acidic proton, we noticed that both anions produced essentially identical fragments corresponding to fatty acid anions at *m/z* 255 and 281. PAGPE fragments due to the neutral loss of fatty acids were also observed. As PAGPE has a protonated ammonium group, which can readily protonate the leaving fatty acid anion, neutral loss of a fatty acid would serve as a better indicator than formation of a fatty acid anion in this type of lipids for assigning fatty acyl to the sn-1 or sn-2 positions. The *m/z* 1134 PAGPE anion produced far more *m/z* 852 fragment due to the neutral loss of 282 amu (18:1) fatty acid than the *m/z* 878 fragment due to the loss of 256 amu (16:0) fatty acid, which appeared to support our assignment of (18:1) fatty acyl group to the central hydroxyls of the two glycerol moieties.

PAGPE co-migrated with LCL in the 1D-TLC separation. 2D-TLC separation with a second acidic mobile phase successfully resolved these two types of lipids (Fig. 5B). Lipids recovered from silica gels at the florescent spot corresponding to PAGPE also produced large amounts of *m/z* 1136 protonated PAGPE ion as well as *m/z* 1158 sodiated PAGPE ion. We therefore acquired and manually interpreted MS/MS spectra of these two cations.

**Figure 6:**
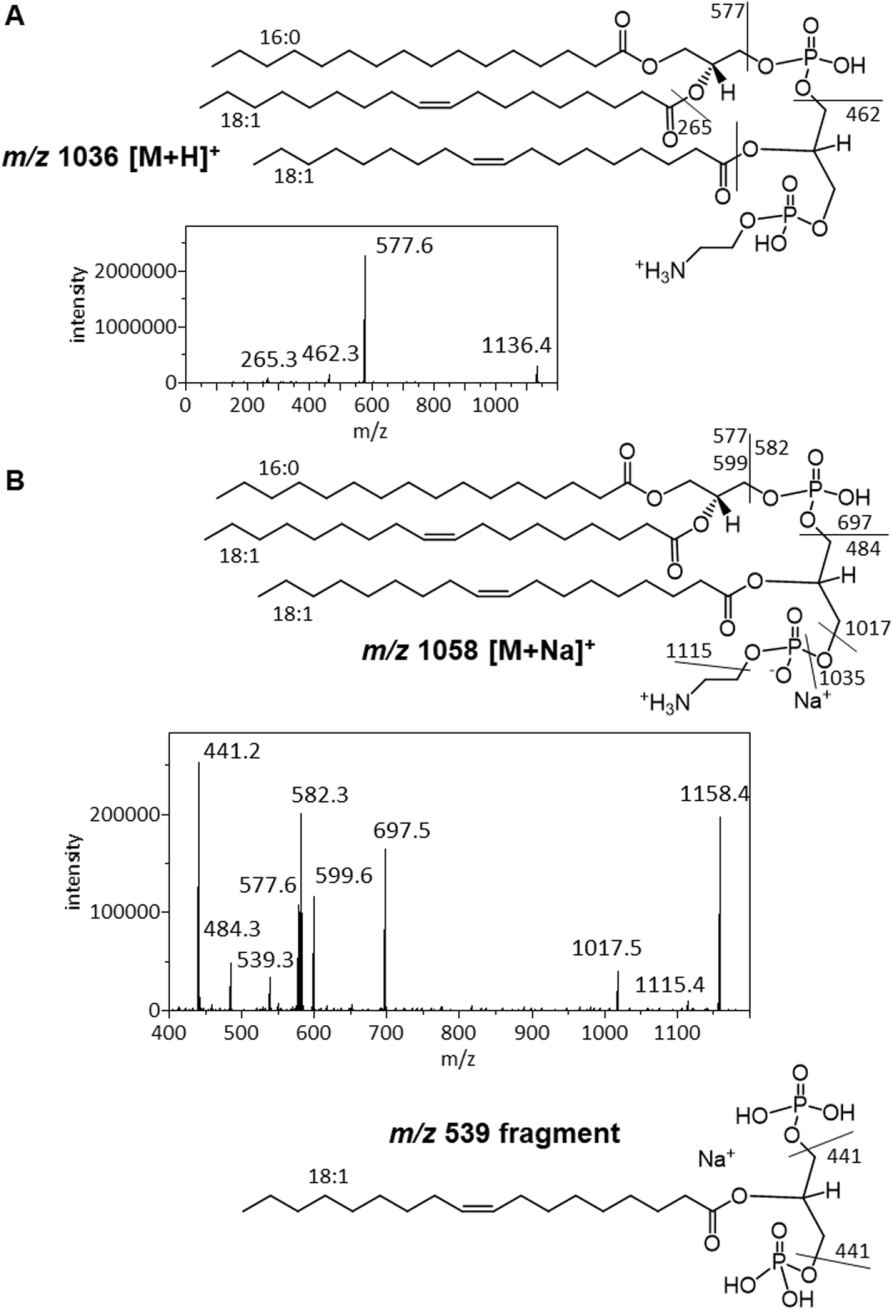
Dissociation and MS/MS spectrum of PAGPE cations. (**A**) Dissociation and MS/MS spectrum of the *m/z* 1136 protonated ion. (**B**) Dissociation and MS/MS spectrum of the *m/z* 1158 protonated ion. Two magnified areas of the spectrum are also shown.

As shown in Fig. 6A, only two major fragments of the *m/z* 1136 protonated PAGPE ion were observed. The predominant *m/z* 577 fragment likely corresponds to the most commonly observed neutral loss of the head group resulting in the formation of a headless glycerolipid fragment^17^ equivalent to a dehydroxylated DG ion ([DG-OH]^+^). The *m/z* 577 fragment is consistent with the most commonly observed fatty acid composition of (16:0–18:1) in *A. radioresistens* lipids. On the other hand, the *m/z* 462 ion likely corresponds to the neutral loss of PA (674 amu) which would produce the [lyso-PE-OH]^+^ ion with a fatty acyl composition of (18:1), and therefore supports the assignment of a (18:1) fatty acyl to the central glycerol of PAGPE.

The sodiated PAGPE cation at *m/z* 1158 provided far more fragmentation information (Fig. 6B) than the *m/z* 1136 protonated ion. The [DG-OH]^+^ and [DG-H_2_O + Na]^+^ twin peaks at 577 and 599 are consistent with the fatty acid composition of (16:0–18:1)as does the *m/z* 697 [PA + Na]^+^ fragment. On the other side of this molecule, the *m/z* 582 fragment is likely due to neutral loss of the 576 amu dehydrated DG, while the *m/z* 484 fragment is likely due to the loss of the 674 amu PA as assigned. The larger fragments of *m/z* 1115, 1035 and 1017 likely corresponds to neutral loss of cyclic ethylamine (43 amu), cyclic phosphoethanolamine (123 amu) and phosphoethanolamine (141 amu), respectively. Detection of these three ions support the terminal assignment of the ethanolamine moiety. The *m/z* 539 fragment likely corresponds to the sodiated ion of the mid-section of the lipid molecule which is 2-acyl-glycerol-1,3-diphosphate with a (18:1) fatty acyl group. A further loss of a phosphoric acid (98 amu) from the *m/z* 539 fragment would lead to the production of the *m/z* 441 fragment.

Another emerging lipid species was detected by MS analysis in lipids extracted from cells in the stationary phase. They appeared to co-migrate with APG in TLC experiment (Fig. 1). We acquired MS/MS spectra of two such anions, which matched the fragmentation observed for chemically synthesized NAPE^18^(Supplementary Fig. S9). The ethanolamine tail appeared to be acylated by (16:0) fatty acid (Fig. 4).

### Onset of stationary phase-triggered lipidome adaptation in *A. radioresistens*

We subsequently standardized time points of 11 hours, 35 hours and 7 days for triple-replicated lipidome analysis, with each time point representing late exponential growth phase, early and late stationary phase, respectively. Cells harvested 11 hours after inoculation had an optical density of 1.6 at 600 nm, and a pH of 7.5. Cell growth entered stationary phase a few hours after that and stabilized at an optical density of 1.8 throughout at least 7 days. The pH was 8.5 when harvested 35 hours after inoculation, and 9.0 after 7 days.

As shown in Fig. 1A,B, TLC analysis using two sets of mixed solvents clearly revealed the lipidome adaptation in *A. radioresistens*. The level of total lipids appeared to be the lowest in extracts 7 days after inoculation (Fig. 1A,B). The fluorescence-based estimate of lipid compositions, as well as their standard deviations, is shown in Fig. 1C. The minor APG species did not appear to respond noticeably to the onset of stationary phase. The estimated percentages of PE and PG, the two most abundant species during the exponential growth phase, diminished as cells entered stationary phase. Even more drastic decrease in PE and PG compositions was observed after 7 days of cell culture. The relative abundance of CL and LCL increased noticeably during this transition, which was almost exactly the opposite to that of PE and PG. This TLC-revealed lipid composition did not correlate well with MS analysis of the lipid extracts (Figs 2 and 3), which is likely due to ion suppression in direct-infusion mass spectrometry. LPAGPE was a minor lipid species on the chromatogram (Fig. 1). NAPE co-migrated with another minor species APG, and must be a minor species. Importantly, NAPE and PAGPE were observed only in lipids extracted from bacteria in the stationary phase that was harvested 35 hours or 7 days after inoculation. PAGPE co-migrated with LCL on one-dimensional thin-layer chromatogram using 70:25:4 chloroform/methanol/water (Fig. 1A). The two species were well separated when 70:25:3:1 chloroform/methanol/water/acetic acid was used as the solvent to develop the thin-layer chromatogram (Fig. 1B). All types of these lipids were separated by 2D-TLC (Fig. 5B). The estimated percentage of PAGPE was highest (12% ± 2%) in the lipids extracted from cells harvested 7 days after inoculation.

Since pH of the media tends to rise during prolonged bacterial culture, we also investigated potential effect of pH. Lipids extracted from the bacteria during exponential growth phase at pH 7.5 and 9.0 were similar. Composition of lipids extracted from the bacteria during stationary phase at pH 7–8 and pH 9.0 were similar as well. For instance, PAGPE was only detected in lipids of bacteria in stationary phase regardless of pH values of the media (Fig. 3 and Supplementary Fig. S11).

To attempt to better define the major groups of nutrients which became depleted during stationary phase, we measured densities of stationary phase cells cultured in LB media supplemented with 5 g/L D-glucose, casamino acids, or Na_2_HPO_4_. All the cultures became alkaline with pH increased to ~9.0 during stationary phase after 48 h. Negligible cell density changes were observed in replicated cultures supplemented with glucose (−2% to 1% change in cell density) or phosphate (−4% to 2% change in cell density). On the other hand, supplementation with casamino acids resulted in ~40% (38% to 43%) increase in cell density.

## Discussion

### Triglycerides

The presence of TG in bacteria, though not as ubiquitous as in eukaryotic organisms, is not rare. They have been frequently found in some Gram-positive genera in the order Actinomycetales including *Mycobacterium, Streptomyces, Rhodococcus* and *Nocardia*^19^. TG has also been found in some Gram-negative bacteria, especially *Acinetobacter* species^19^. *A. radioresistens* as well as other *Acinetobacter* species are known to produce highly acylated bioemulsifiers^20^. It is possible that the TG serve as a fatty acid reserve for producing such emulsifiers. TG appeared to be as abundant in lipids extracted from the bacteria during exponential and stationary phases. This is consistent with the observation that the carbon source was not depleted in the media.

### Common glycerophospholipids

APG is almost exclusively found in bacteria including *Acinetobacter* species^21,22^. As seen in other bacteria, the third fatty acyl is likely transferred to the terminal hydroxyl of the PG head group to form APG^21,23^. It has rarely been observed as a major lipid^24^. PE, PG, and CL are the common major components of bacterial membrane. It is somewhat surprising that LCL was observed as a major lipid, especially in the stationary phase. These five lipids extracted from *A. baumannii* in the stationary phase have recently been characterized by TLC and MS^25^. In comparison, fatty acid compositions of these lipids in *A. baumannii* and *A. radioresistens* are very similar, which is consistent with their evolutionary relationship. In both *Acinetobacter* species, the predominant fatty acid composition is (16:0–18:1), which corresponds to the most abundant PE, PG, APG, LCL and CL single ions observed at *m/z* 716, 747, 985, 1165 and 1403, respectively.

### Phosphatidyl-acyl-glycero-phosphoethanolamine

PAGPE appears to be a novel lipid species we observed only in the stationary phase of *A. radioresistens* (Fig. 1B,C). PAGPE production coincided with lipidome transition from a PE and PG-dominated state to a CL and LCL-dominated state. It is worth noting, transition into a CL-rich state is a known common characteristic of model bacteria *E. coli* and *B. subtilis* in nutrient-depleted stationary phase^4,5^. As the lipidome transitions into a CL and LCL-dominated state while PE content diminishes, electrostatic destabilization of the membrane would be expected to increase due to repulsion between anionic head groups of CL and LCL. Unlike PE, PAGPE has a compatible overall architecture to that of CL and especially LCL. We therefore speculate that PAGPE may intercalate amongst CL and LCL and serve as a potential membrane stabilizer to alleviate repulsive forces between phosphate groups, and thus may serve as an important component of bacterial lipidome when this bacterium switch to a slow-growing or growth-arrested state during prolonged nutritional stress.

### N-acyl-phosphatidylethanolamine

NAPE is a lipid found in plants and animals^26^. To the best of our knowledge, NAPE has not been reported as a bacterial metabolite. It appeared to be produced by *A. radioresistens* only during the stationary phase. Its tri-acylated structure appears to be more compatible in size to APG but with a polar amide group, which, like PAGPE, may also contribute to stabilization of the predominantly negative lipids in the membrane.

### Starvation-triggered production of tri-acylated glycerophospholipids

Except for APG, production of three out of the four tri-acylated phospholipids in *A. radioresistens* appeared to be triggered or ramped up at the onset of stationary phase (Fig. 1C). It is not clear which factor(s) during the transition into stationary phase triggered such biosynthesis. Nevertheless, the growth phase-dependent production of these lipids clearly indicates adaptation in anabolic activities when cell growth is slowed or arrested. Most bacteria must endure periods of nutrient depletion or even dehydration.

### Correlation between cell morphology transition and lipidome change

Strategies evolved in bacteria to cope with environmental challenges could aid in the development of more effective bactericidal agents. The study of slow-growing or growth-arrested bacteria has recently become important as some characteristics of bacteria in stationary phase resembled those seen in bacterial persister cells that can withstand antibiotic treatment and cause relapsing infections^27^. Nutritional stress, along with many other environmental stressors, is in fact an important factor that triggers the formation of persister cells.

*Acinetobacter* species tend to be rod shaped during growth and become shorter, narrower and more spherical upon nutrient depletion^28^. This phenomenon appears to be shared by many bacteria including *Escherichia coli*^29^ and *Pseudomonas aeruginosa*^30^. The increased curvature in the bacterial membrane during stationary phase is consistent with the observed increase in the level of CL which is a curvature-seeking lipid in model organisms *E. coli* and *B. subtilis*^31–33^. Unlike *E coli*, which has all three known types of bacterial CL synthases ClsA, ClsB, and ClsC^34^, only one type of CL synthase (GenBank: KX263725) was identified in all sequenced *Acinetobacter* species by BLASTP^35^ with ~40% sequence identity to *E. coli* ClsC. Over-expression of *A. radioresistens* ClsC (ArClsC) in BL21(DE3) cells significantly increased CL content in the host bacteria (Supplementary Fig. S12). The *E. coli* ClsC protein (previously named YmdC) is a stationary phase membrane remodeling enzyme which converts PG and PE into CL and ethanolamine^36^. Our observation suggests that ArClsC may function similarly. On the other hand, the biosynthetic pathway of PAGPE has yet to be characterized. The inner and outer leaflets of the cell membrane have opposite requirements for the ratio of polar head group and alkyl tail. CL has a smaller footprint of the head group as compared to its alkyl tail, which makes CL an ideally shaped lipid to localize in the inner leaflet of bacterial membrane. On the other hand, the head/tail ratios of LCL and PAGPE appear to be larger than that of CL. As such, the inner leaflet is unlikely to be the ideal localization for these two tri-acylated lipids. We hypothesize that LCL and PAGPE may be ideal candidates for localization in the outer leaflet of bacterial membrane, especially during stationary phase when the curvature is more pronounced for most part of the bacterial membrane.

## Methods

### Bacterial strain and cell culture

The strain of *A. radioresistens* was an accidental isolate in the lab as a chloramphenicol-resistant bacterium. This organism was identified by matrix-assisted laser desorption ionization – time of flight (MALDI-TOF) mass spectrometry using the bioMerieux Vitek MS system in the Clinical Microbiology Laboratory, Saskatoon Health Region. Bacterial cell suspensions in Luria-Bertani broth (LB) supplemented with 20% glycerol were stored at −80 °C. Autoclaved 10 mL of LB media supplemented with 35 µg/mL of chloramphenicol was inoculated with the bacterial freezer stock and placed in an environmental shaker for overnight incubation. All cell incubations were carried out at 37 °C and 220 RPM. A glass flask with 50 mL LB media was inoculated with 50 μL of the overnight culture and incubated for 11 h, 35 h, or 7 d to reach late exponential growth phase, early stationary phase, and prolonged stationary phase, respectively. After measuring pH with pH paper and cell density by absorbance at 600 nm, the cell suspension was transferred to a disposable 50-mL centrifuge tube, supplemented with 0.75 mL of 1.0 M sodium acetate buffer at pH 5.2, and centrifuged at 4,000 rpm for 16 min at 4 °C using a Beckman-Coulter TS-5.1–500 rotor in Allegra 25 centrifuge. The wet cell pellet was rinsed for 5 s, twice, with ice-chilled ddH_2_O before resuspending in 0.5 mL ddH_2_O and 2.0 mL methanol for lipid extraction.

### Lipid extraction

HPLC-grade organic solvents (Fisher Scientific, Ottawa, ON, Canada) and distilled deionized water were used throughout the experiment. Bacterial lipids were extracted following the Bligh-and-Dyer method^15^ with glass syringes, glass tubes, and ice-chilled solvents throughout the extraction process as previously described^37^. After adding chloroform for monophasic extraction and eventual phase separation enhanced by centrifugation at 1,300 rpm for 5 min, the initial chloroform-rich phase was collected and supplemented with 0.5 mL 0.5 M sodium chloride (NaCl). After shaking by hand for 1 min, phase separation was again enhanced by centrifugation at 1,300 rpm for 5 min. The final chloroform-rich phase, approximately 4.2 mL, was collected for storage at −80 °C.

### Lipid profiling by Mass Spectrometry

The lipid sample was diluted with methanol supplemented with 5 mM NH_4_OH prior to direct infusion at a rate of 0.9 mL/h into a QTRAP 4000 LC-MS/MS (Applied Biosystems, Foster City, CA, USA) mass spectrometer equipped with a Turbo V Ion Spray electrospray ionization (ESI) source. Three-fold dilution in methanol was found to maximize signals while suppressing signals of adducts such as PG dimer and PG-PE heterodimer. Optimal electrospray ionization was achieved at a temperature of 400 °C and ionization voltage of −4500 V or +5500 V. The precursor scans were acquired with collision energies set at −65 or +45 eV in negative or positive mode, respectively. The SCIEX Analyst 1.6 software was used to acquire and export averaged mass spectra in text format. Mass spectra were also analyzed by Mass++ 2.7.4 software^38^. The MS figures were generated by Mass++ 2.7.4. Chemical structures were drawn using PerkinElmer ChemDraw prime 15.101.144.

### Tandem mass spectrometry

The targeted MS/MS spectra were acquired using the QTRAP 4000 mass spectrometer in Enhanced-Product-Ion mode. While fixing an energy spread of 10 eV, collision energy in the ion trap was optimized in the range of 30 to 80 eV to acquire the most informative MS/MS spectra. In negative mode, the collision energy was typically 65 eV for single anions and 40 eV for double anions. In positive mode, the typical collision energy was 45 eV.

### Lipid analysis by thin-layer chromatography

A volume of 200 µL of lipid sample, approximately 5% of the lipid extract from 50-mL bacterial culture, was transferred to an Agilent glass sample vial, dried for 10 min in vacuum, and resuspended in 30 µL of 4:1 chloroform/methanol. Then a glass spotting capillary tube was used to spot the sample 1.8 cm above the bottom edge of an EMD Millipore TLC plastic sheet cut to a height of 10 cm. After drying in air at an ambient temperature of 21 °C for 20 min, the TLC sheet was placed into a TLC chamber pre-equilibrated with a mixed solvent of chloroform/methanol/water (70:25:4) or chloroform/methanol/water/acetic acid (70:25:3:1). After 25 min of TLC development, the TLC sheet was removed from the TLC chamber and dried for 20 min at ambient temperature. For 2D-TLC analysis, the dried TLC sheet was rotated 90 degrees and placed into a TLC chamber pre-equilibrated with a mixed solvent of chloroform/methanol/water/acetic acid (70:25:4:1), developed for another 25 min, and dried for 20 min. The dried TLC sheet was sprayed with 0.02% primuline (Sigma-Aldrich) solution in 80:20 acetone/water, and then dried in air at ambient temperature for an hour. The fluorescent image was recorded with a Syngene G:BOX system. The silica gels at each representative fluorescent band were lifted with a metal spatula and suspended in 200 µL chloroform in an Agilent glass sample vial and stored at −20 °C. 400 µL methanol was added to the suspension right before MS analysis. After shaking by hand for 10 s followed by sedimentation of silica gel for 2 min, the clarified sample was injected into the mass spectrometer for analysis. MS scan, precursor scan, neutral loss scan, and MS/MS spectra were acquired to assign lipid composition in each TLC fluorescent band. After baseline subtraction, fluorescence intensities of lipid bands on thin-layer chromatograms were integrated using GelAnalyzer version 2010a.

## Electronic supplementary material

Supplementary Info
