# Supplementary material for: Nutrient depletion-induced production of tri-acylated glycerophospholipids in Acinetobacter radioresistens
Source: Sci Rep. 2018 May 10;8:7470. doi: 10.1038/s41598-018-25869-9 (PMC5945596; doi:10.1038/s41598-018-25869-9)
Supplement: Supplementary file 1 — Supplementary Info [file 41598_2018_25869_MOESM1_ESM.pdf]

Nutrient depletion-induced production of tri-acylated glycerophospholipids in *Acinetobacter radioresistens*

Yu Luo[*1], Muhammad Afzal Javed[2], Harry Deneer[3,4], Xialu Chen[1]

[1] Department of Biochemistry, College of Medicine, University of Saskatchewan, Saskatoon, Saskatchewan, Canada

[2] Department of Microbiology and Immunology, College of Medicine, University of Saskatchewan, Saskatoon, Saskatchewan, Canada

[3] Department of Pathology and Laboratory Medicine, College of Medicine, University of Saskatchewan, Saskatoon, Saskatchewan, Canada

[4] Molecular Microbiology Laboratory, Division of Clinical Microbiology, Saskatoon Health Region, Saskatoon, Saskatchewan, Canada

*To whom correspondence should be addressed: Prof. Yu Luo, Telephone: 1 306 966-4379; Fax: 1 306 966-4390; Email: yu.luo@usask.ca

Supplementary data

Introduction: Here we mainly present the molecular dissociation mechanisms, tandem MS spectra, and tables of ionic species with manually assigned fatty acid compositions. The original TLC chromatograms are shown as well. Manual interpretation of MS / MS spectra acquired with the 4000 QTRAP system was mainly based on the following three articles:

Hsu F.F., Turk J., Rhoades E.R., Russell D.G., Shi Y. and Groisman E.A. Structural characterization of cardiolipin by tandem quadrupole and multiple-stage quadrupole ion-trap mass spectrometry with electrospray ionization. J Am Soc Mass Spectrom. 2005.**16(4)**: 491-504.

Murphy, R.C. and P.H. Axelsen, Mass spectrometric analysis of long-chain lipids. Mass Spectrom Rev, 2011. **30(4)**: 579-99.

Coulon, D. and Bure, C. Acylphosphatidylglycerol (acyl-PG) or N-acylphosphatidylethanolamine (NAPE). J. Mass Spectrom, 2015. **50**: 1318-20

We also present a 2D-TLC chromatogram which gave a better separation than 1D-TLC.

Abbreviations: L – lyso, as in LPA, LPG, LPE, LCL and LPAGPE; FA – fatty acid; TG – triglyceride; MG – monoglyceride; DG – diglyceride; PG – phosphatidylglycerol; APG – acyl-phosphatidylglycerol; NAPE – N-acyl-phoshatidylethanolamine; PAGPE – 1-phosphatidyl-2-acyl-glycero-3-phosphoethanolamine; PE – phosphatidylethanolamine; PA – phosphatic acid; CL – cardiolipin.

In negative mode, most glycerophospholipids give off strong fragment signals corresponding to fatty acid anions. Typically, though not always, signal of fatty acid located at the sn-2 position is stronger than that at the sn-1 position. Notable exception to this rule has been observed for singly charged cardiolipin anion. This enabled us to make position-specific fatty acid assignments to lipid precursor ions based on this weak and strong pairing. Lipid precursor ions are often composed of isobaric isomers. Occurrence of multiple weak and strong pairs of fatty acid anions enabled us to assign major and minor isomers based on signal intensity.

As expected for glycerophospholipids, all the presented MS / MS spectra harbored low levels of *m/z* 79, 97 anions corresponding to phosphite and phosphate anions, respectively, as well as the *m/z* 153 cyclo-glycerol-phosphate anion. These three ions will no longer be singled out in the supplementary figures.

Neutral loss of a fatty acid or ketene results in twin peaks separated by *m/z* 18. Only one of the two related reactions are annotated in the dissociation mechanism when it is too crowded to show both.

We have acquired MS / MS spectra of selected lipid precursor ions with ion count larger than 1 x 10^5, a practical threshold for generating meaningful MS / MS spectra with the QTRAP system. The predominant fatty acid compositions are tabulated for these precursors.

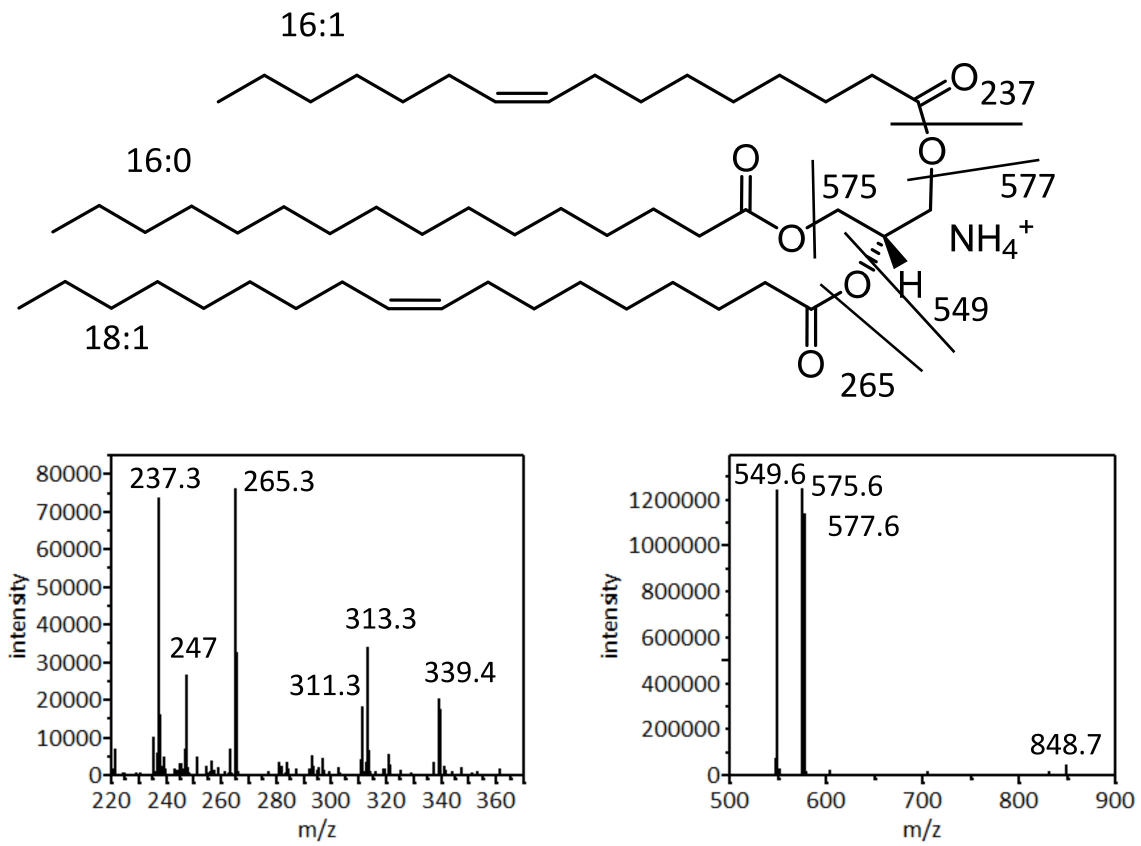

Figure S1: Dissociation mechanism and MS/MS spectrum of *m/z* 848 triglyceride ammonium adduct [TG + NH4]$^+$.

Unlike several *Bacillus* and *Staphylococcus* bacteria, which have abundance of DG, *A. radioresistens* was observed to be rich in TG instead. The typical MS/MS spectrum of the [TG+NH4]$^+$ cation at *m/z* 848 is shown above in Figure S1. [DG-OH]$^+$ ions at *m/z* 577, 575 and 549 correspond to [DG-OH]$^+$ ions as the result of neutral losses of (16:1), (16:0) and (18:1) fatty acid ammonium salts, respectively. Consistent with the TG structure shown, [MG-OH]$^+$ ions at *m/z* 311, 313 and 339, which are likely produced by successive losses of a ketene and a fatty acid ammonium salt, correspond to fatty acid compositions of (16:1), (16:0) and (18:1), respectively. Cations at *m/z* 237 and 265 also correspond to [FA-OH]$^+$ ions of the two unsaturated fatty acids of compositions (16:1) and (18:1), respectively.

Fatty acid compositions of TG ammonium adduct ions of adequate abundance for MS/MS analysis were assigned, as listed in table S1. The three fatty acids are listed with ascending order in size. Their positions in the triglyceride structure are uncertain. The four most abundant TG adduct cations are highlighted in bold.

**Table S1. $[TG + NH4]^+$ (*m/z*)**	**fatty acid composition**
822	16:1-16:0-16:0
**848**	**16:1-16:0-18:1**
**850**	**16:0-16:0-18:1**
864	16:0-17:0-18:1
**876**	**16:0-18:1-18:1**
**878**	**16:0-18:1-18:0**
890	17:0-18:1-18:1
892	17:0-18:1-18:0
904	18:1-18:1-18:0

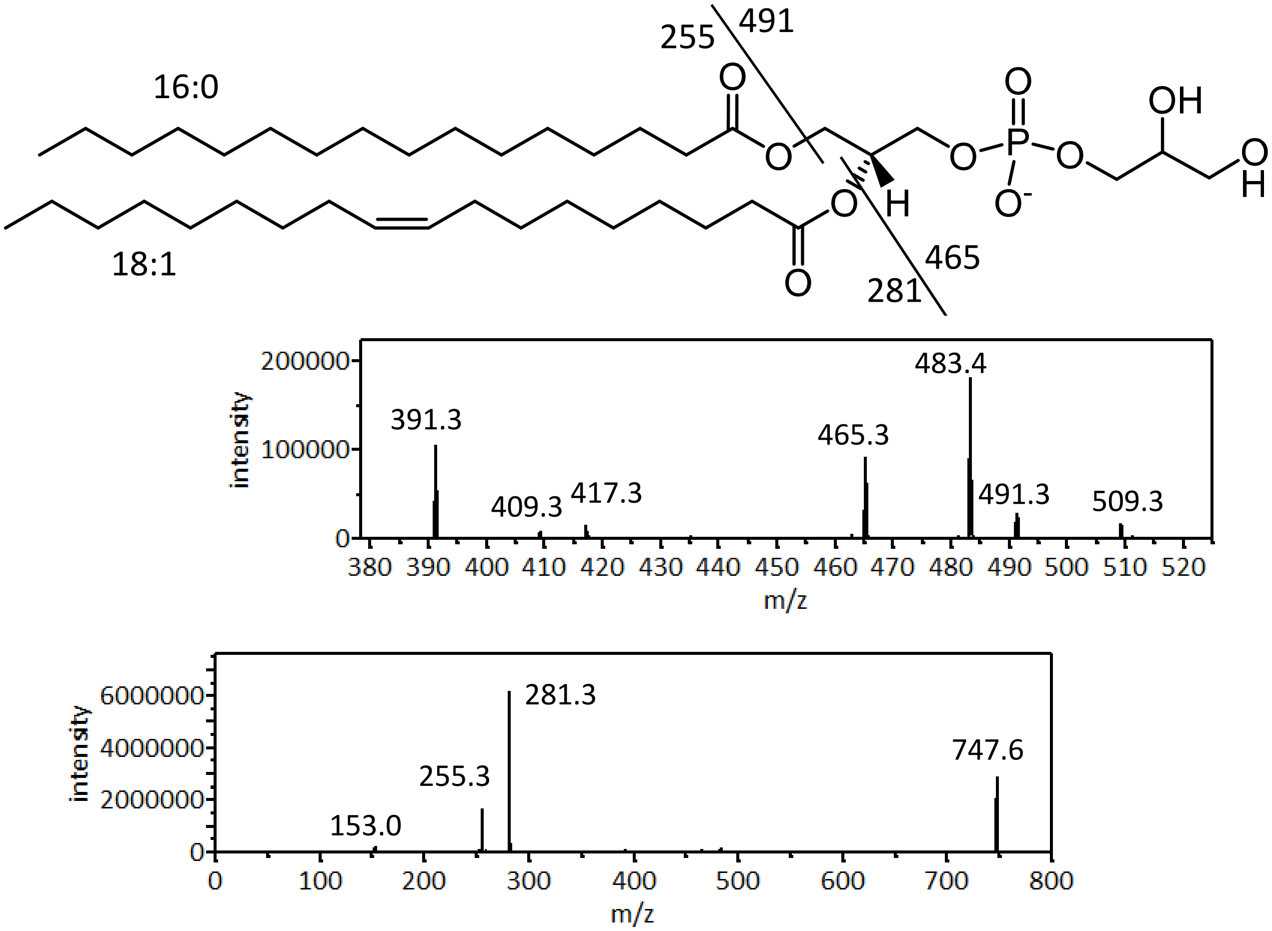

Figure S2: Dissociation mechanism and MS/MS spectrum of the *m/z* 747 (16:0-18:1) [PG - H]$^-$.

PG anions were by far the most abundant in negative MS spectrum. MS/MS spectrum of the predominant *m/z* 747 PG anion is shown above in Figure S2. The more intense fatty acid anion at *m/z* 281 indicates a (18:1) fatty acyl at the sn-2 position. The less intense *m/z* 255 indicates a (16:0) fatty acyl at the sn-1 position. Neutral loss of one of these two fatty acids or their ketene residues would result in the *m/z* 465 / 483 pair and the *m/z* 491 / 509 pair. Peaks at *m/z* 391 and 417 correspond to (16:0) and (18:1) cyclic LPA anions caused by the loss of cyclo-glycerol and one fatty acid.

Since PG anions were the most abundant, we were able to acquire meaningful MS/MS spectra of several of such anions and assigned their fatty acid compositions, as listed in table S2. These compositions serve as a near complete survey of fatty acid compositions in glycerophospholipids found in *A. radioresistens*. The two fatty acids are listed with composition of the fatty acyl at the sn-1 position in the front. The fatty acyl positions were based on the observation that the fatty acid anion dissociated from the sn-2 position is more abundant than that from the sn-1 position. The *m/z* 733 anions were likely a mixture as less intense isobaric pairs of fatty acid anions were also observed. The *m/z* 747 anion was by far the most abundant, followed by the *m/z* 745, 719, 773 and 775 ions. All four are highlighted in bold.

**Table S2. $[PG - H]^-$ (*m/z*)**	**fatty acid composition**
**719**	**16:0-16:1**
733	16:0-17:1, 17:0-16:1, 15:0-18:1
**745**	**16:1-18:1**
**747**	**16:0-18:1**
759	17:1-18:1
761	17:0-18:0
**773**	**18:1-18:1**
**775**	**18:0-18:1**

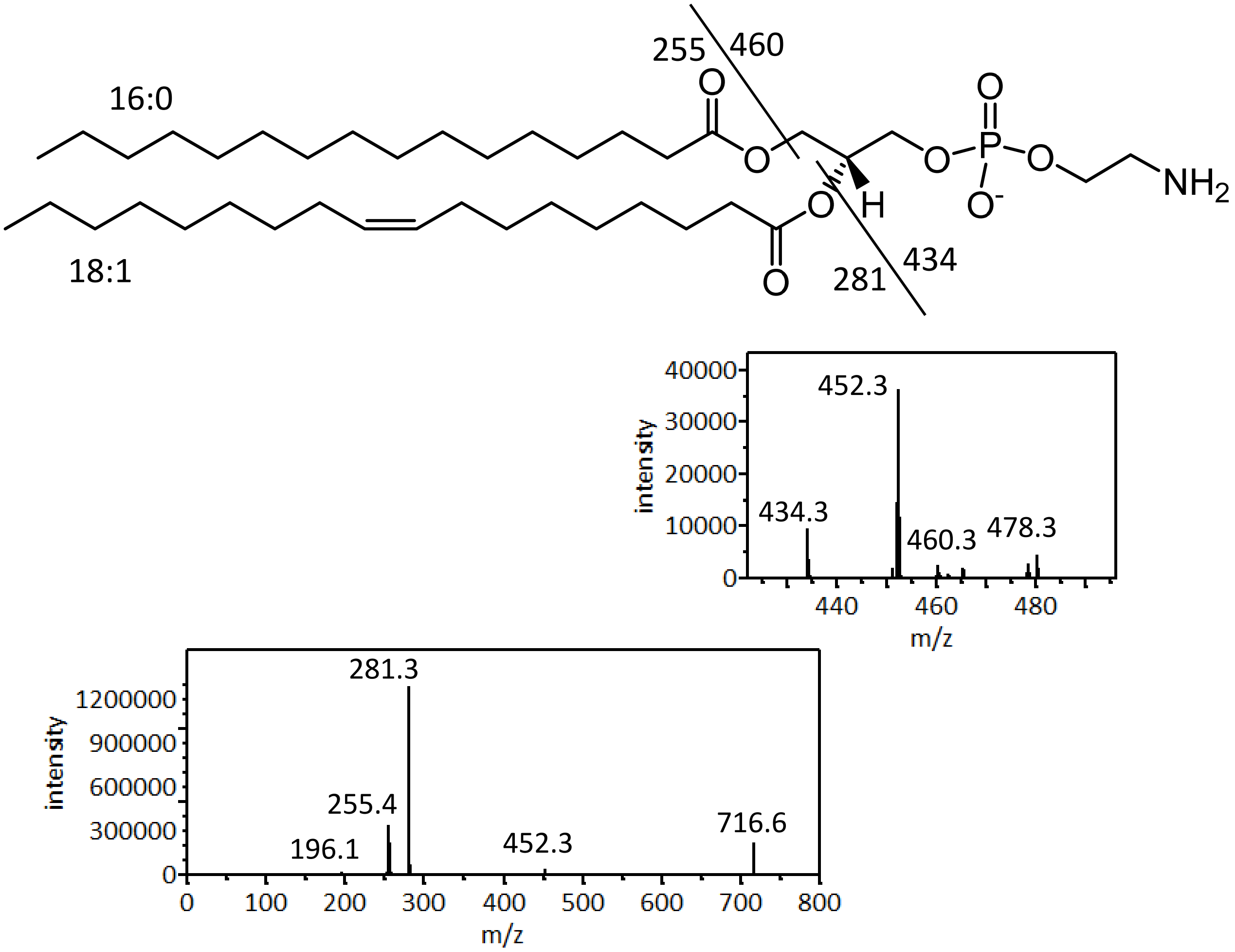

Figure S3: Dissociation mechanism and MS/MS spectrum of *m/z* 716 (16:0-18:1) [PE - H]$^-$.

During the exponential growth phase, PE was as abundant as PG in thin-layer chromatogram. However, PE anions were less abundant than the PG anions due to the zwitterionic structure of PE. MS/MS spectrum of the predominant PE anion at *m/z* 716 is shown above in Figure S3. Signature fragments of PE at *m/z* 140 and 196 were observed, corresponding to phosphoethanolamine and cyclic glycerophosphoethanolamine anions, respectively. The difference in intensity of (16:0) and (18:1) fatty acid anions at *m/z* 255 and 281 indicated their sn-1 and sn-2 positions, respectively. Neutral loss of one of these fatty acids or in their respective ketene forms would produce the *m/z* 434 / 452 pair and the *m/z* 460 / 478 pair.

Fatty acid compositions of major PE anions were assigned based on MS/MS spectra, as listed in table S3. The two fatty acids are listed with composition of the fatty acyl at the sn-1 position in the front. Like PG, the fatty acyl assignments for PE were also based on the observation that the fatty acid anion dissociated from the sn-2 position is more abundant than that from the sn-1 position. The PE anion at *m/z* 716 (highlighted in bold) was by far the most abundant, followed by the *m/z* 714, 688 and 744 ions.

**Table S3. [PE - H]$^-$ (*m/z*)**	**fatty acid composition**
686	16:1-16:1
**688**	**16:0-16:1**
**714**	**16:1-18:1**
**716**	**16:0-18:1**
742	18:1-18:1
**744**	**18:0-18:1**

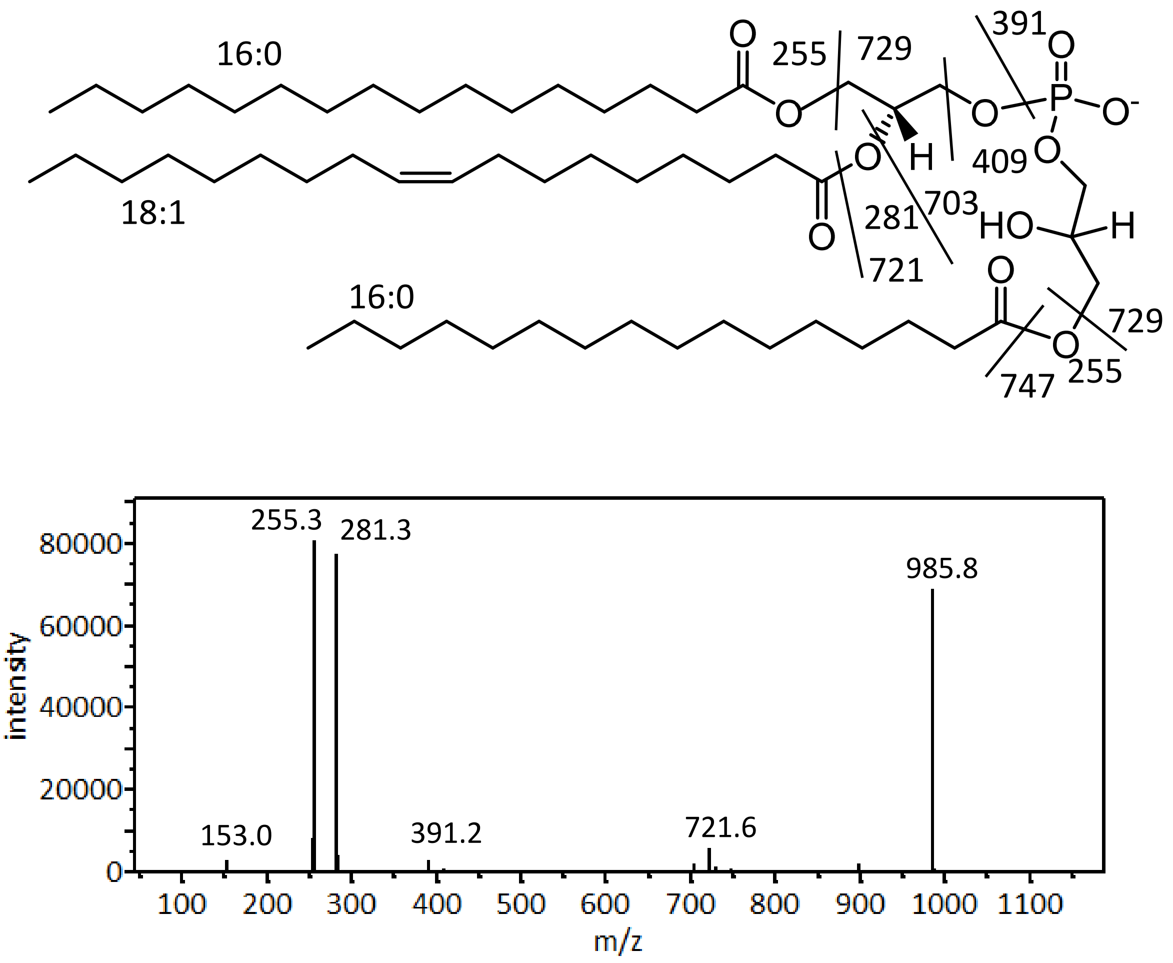

Figure S4: Dissociation mechanism and MS/MS spectrum of *m/z* 985 [APG - H]$^-$.

In addition to PG and PE, we also observed lower quantities of APG. The MS/MS spectrum of the most abundant APG anion at *m/z* 985 is shown in Figure S4. Similar to dissociation of PG, bond breakage at either side of the three ester bonds would result in formation of ketene or fatty acid, leading to the major fragments observed. The positional assignment for each fatty acyl is less certain than for PG and PE anions. The *m/z* 391 / 409 anions correspond to (16:0) cyclic / linear LPA anion. Our tentative assignment of this structure is largely based on the most abundant PG configuration of (16:0-18:1), and that the third fatty acyl has been observed at the sn-3' position in APG extracted from other bacteria.

Fatty acid compositions of major APG anions were assigned based on MS/MS spectra, as listed in table S3. The two fatty acids are listed with composition of the fatty acyl at the sn-1 position in the front. The 985 and 1011 anions were more abundant than others.

**Table S4. [APG - H]$^-$ (*m/z*)**	**fatty acid composition (sn-1, sn-2, sn-3')**
955	16:0-16:1-16:1
957	16:0-16:0-16:1
983	16:0-16:1-18:1
**985**	**16:0-18:1-16:0**
1009	16:1-18:1-18:1
**1011**	**16:0-18:1-18:1**
1037	18:1-18:1-18:1

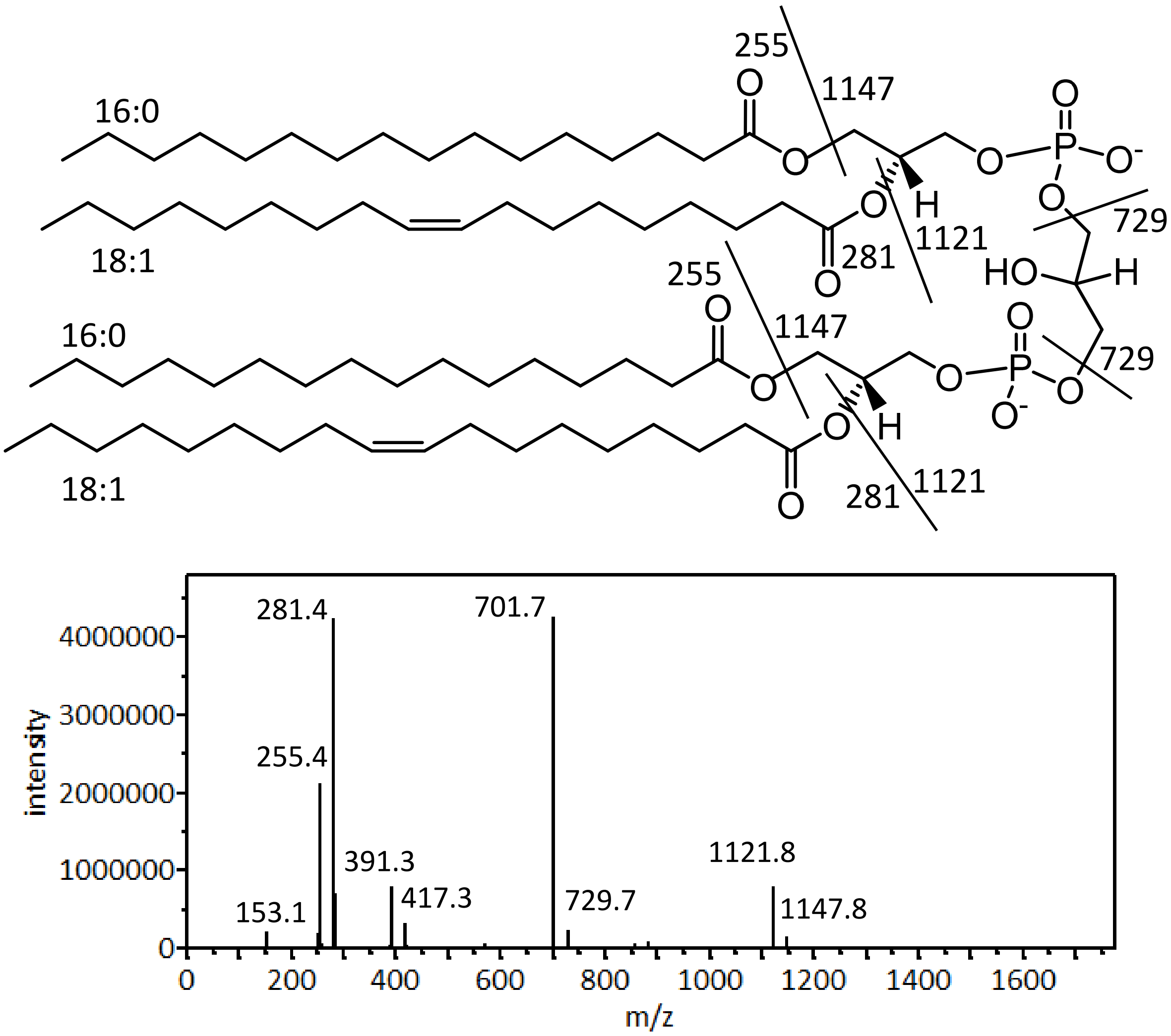

Figure S5A: Dissociation mechanism and MS/MS spectrum of *m/z* 701 precursor $[CL - 2H]^{2-}$.

CL was more abundant as cells enter stationary growth phase, and was observed predominantly as double anions. The MS/MS spectrum of the most abundant LCL double anion at *m/z* 701 is shown in Figure S5. As for PG and PE, the (18:1) fatty acid at the sn-2 position was observed as a more intense *m/z* 281 anion than the *m/z* 255 anion (16:0) at the sn-1 position. Loss of either of these two fatty acid anions would produce LCL single anions at 1121 and *m/z* 1147, respectively. The peak at *m/z* 729 corresponds to cyclic PG anion with a fatty acyl composition of (34:1). Cyclic LPA anions with (16:0) and (18:1) fatty acyl groups were observed at *m/z* 391 and 417, respectively.

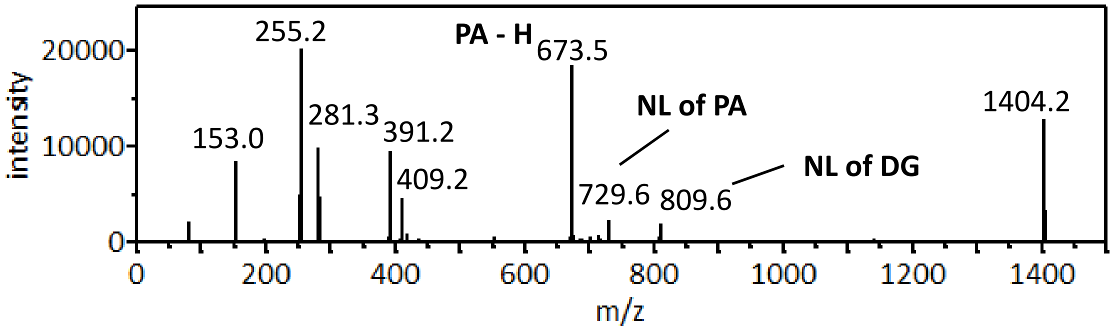

Figure S5B. MS/MS spectrum of *m/z* 1403 precursor of [CL - H]$^-$.

Fatty acid compositions of several major CL single / double anions were assigned based on MS/MS spectra, as listed in table S5. It is worth noting that singly charged CL and LCL anion produces more intense sn-1 fatty acid anion than sn-2 fatty acid anion, which is contrary to PG, PE, as well as doubly charged CL and LCL anions. The two fatty acids are listed with composition of the fatty acyl at the sn-1 position in the front. The fatty acyl positions were based on the observation that the fatty acid anion dissociated from the sn-2 position is more abundant than that from the sn-1 position. Fatty acyl composition of the two glycerol moieties are separated by a slash. The *m/z* 700 and 701 CL double anions were observed to be the most abundant.

**Table S5. [CL - H]$^-$ (*m/z*)**	**fatty acid composition**
1375	16:0-16:1 / 16:0-18:1
1403	16:0-18:1 / 16:0-18:1
1429	18:1-18:1 / 16:0-18:1
**[CL - 2H]$^{2-}$ (*m/z*)**	**fatty acid composition**
673	14:0-16:1 / 16:0-18:1
686	16:1-16:1 / 16:0-18:1
687	16:0-16:1 / 16:0-18:1
**700**	**16:1-18:1 / 16:0-18:1**
**701**	**16:0-18:1 / 16:0-18:1**
713	18:1-18:1 / 16:1-18:1
714	18:1-18:1 / 16:0-18:1

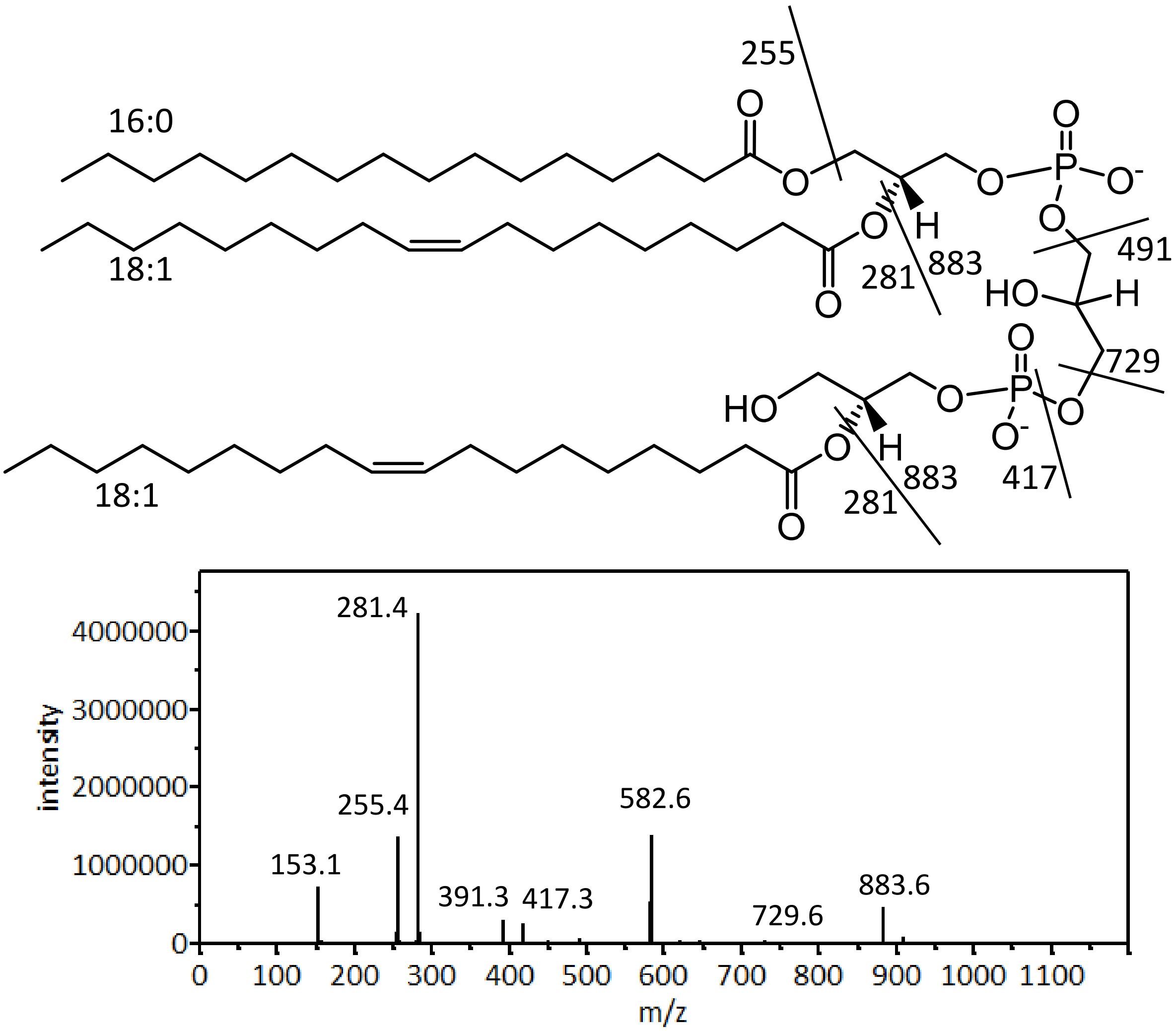

Figure S6A: Dissociation mechanism and MS/MS spectrum of *m/z* 582 precursor of [LCL - 2H]$^{2-}$.

Like CL, LCL was also was also more abundant in the stationary phase, and was observed predominantly as double ions. The MS/MS spectrum of the most abundant LCL double anion at *m/z* 582 is shown in Figure S6. The (18:1) fatty acid at the sn-2 position was observed as a more intense *m/z* 281 anion than the *m/z* 255 one (16:0) at the sn-1 position. Loss of 281 amu anion would produce cyclic dilyso-CL anions at *m/z* 883. Cyclic LPA anions with (16:0) and (18:1) fatty acyl were observed at *m/z* 391 and 417, respectively. The *m/z* 491 ion corresponds to cyclic LPG with a (18:1) fatty acyl group. As for CL, cyclic PG anion at *m/z* 729 suggests a (34:1) fatty acid composition for the DG moiety.

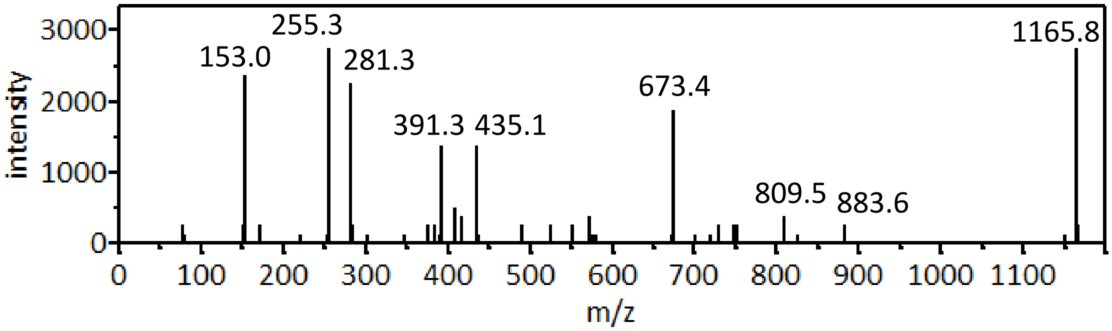

Figure S6B. MS/MS spectrum of *m/z* 1165 precursor of [LCL - H]$^-$.

Fatty acid compositions of three major LCL double anions were assigned based on MS/MS spectra, as listed in table S6. Only one single ion of LCL at *m/z* 1165 was observed in adequate abundance for MS/MS analysis. As for singly charge CL anion, this *m/z 1165* anion produced higher amount of fatty acid anions from the sn-1 position. Fatty acyl composition of the two glycerol moieties are separated by a slash. The second most abundant *m/z* 568 double anions of LCL were likely a mixture. Comparisons were made with the dominant CL species. It appears that the major LCL species are produced by the hydrolytic loss of a saturated fatty acid predominantly located at the sn-1 position, implying activities of a bacterial phospholipase A1.

**Table S6. [CL - H]$^-$ (*m/z*)**	**fatty acid composition**
1165	16:0-18:1 / -18:1
**[CL - 2H]$^{2-}$ (*m/z*)**	**fatty acid composition**
567	16:1-18:1 / -16:1 or 16:1-16:1 / -18:1
**568**	**16:0-18:1 / -16:1 or 16:0-16:1 / -18:1**
575	16:0-17:1 / -18:1 or 16:0-18:1 / 17:1
**581**	**16:1-18:1 / -18:1**
**582**	**16:0-18:1 / -18:1**
589	17:0-18:1 / -18:1
**595**	**18:1-18:1 / -18:1**

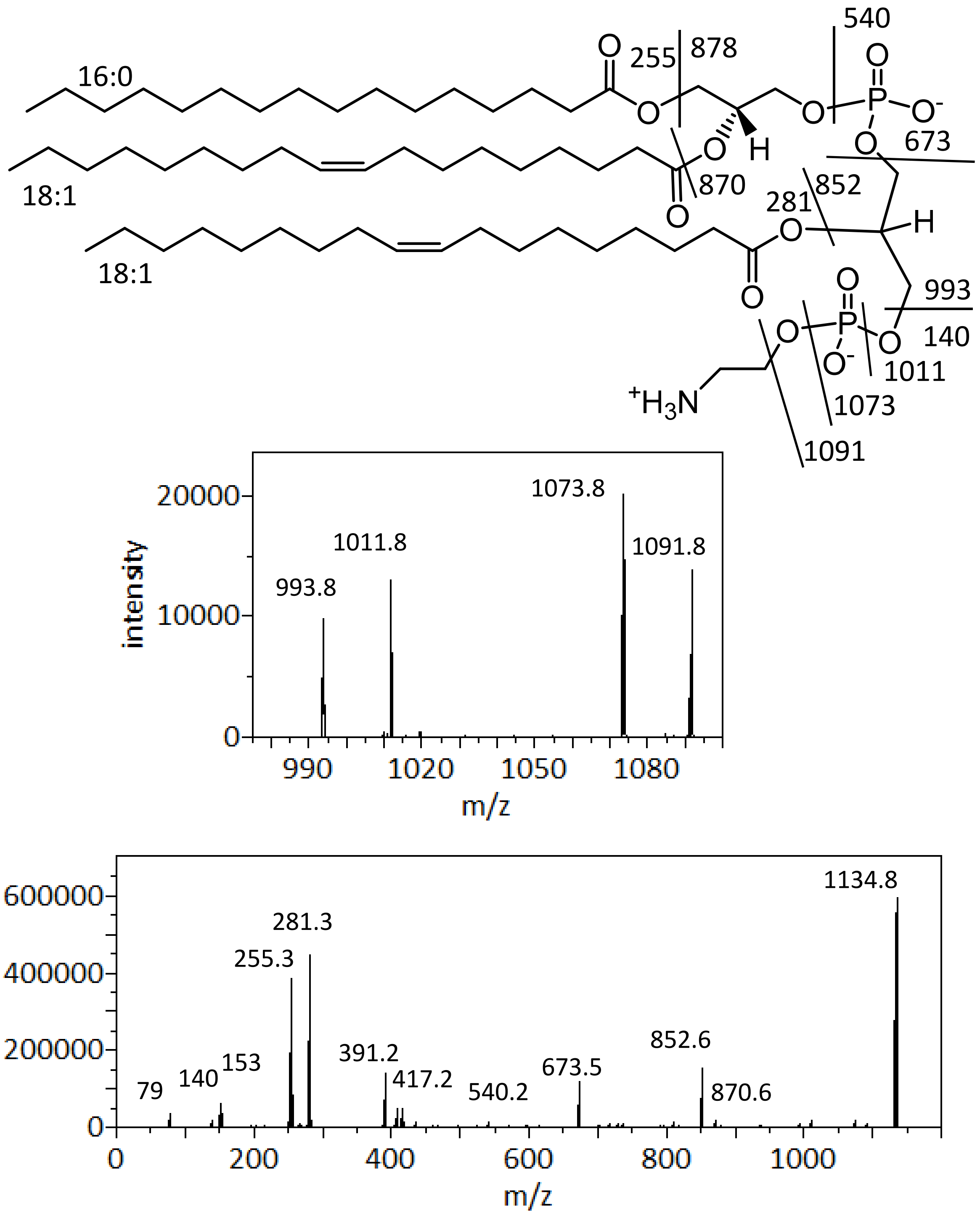

Figure S7A: Dissociation mechanism and MS/MS spectrum of *m/z* 1134 [PAGPE - H]$^-$.

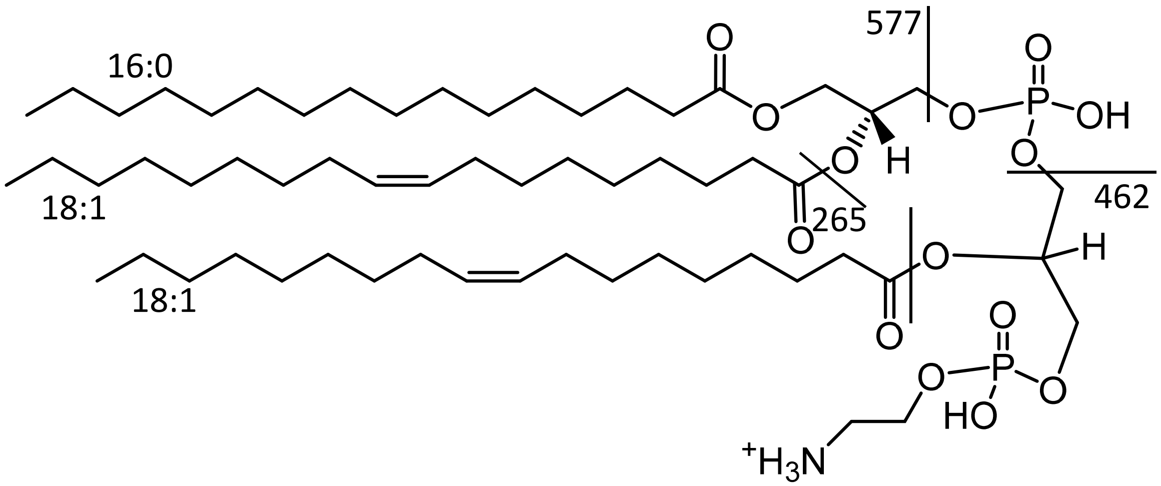

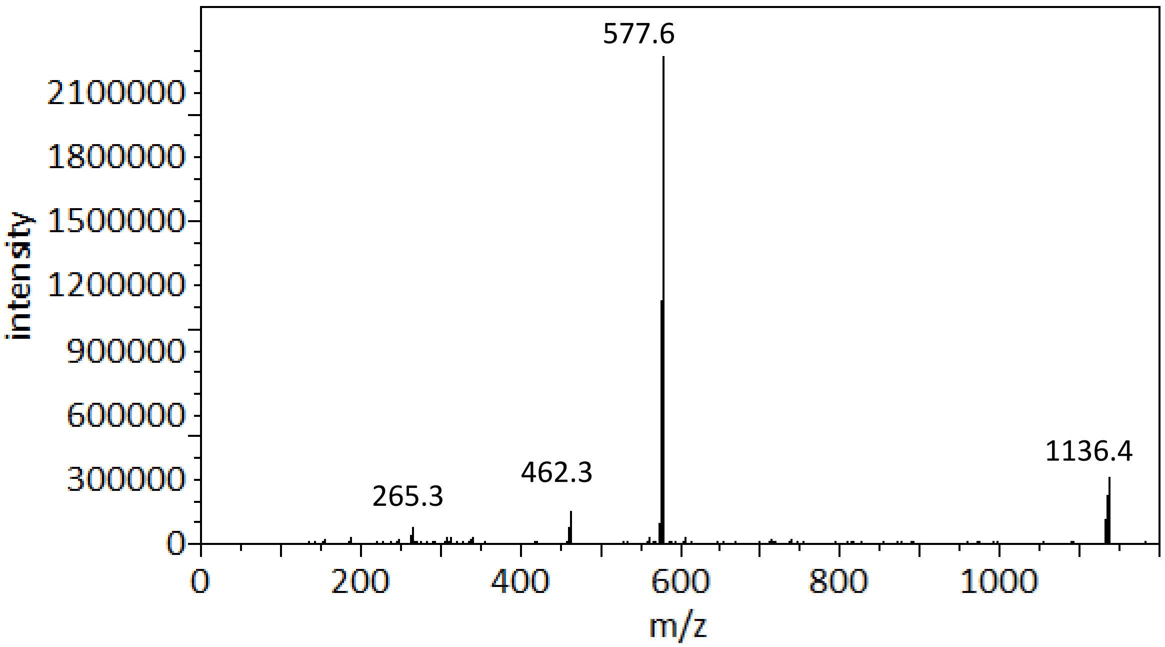

Figure S7B: Dissociation mechanism and MS/MS spectrum of *m/z* 1136 [PAGPE + H]$^{+}$.

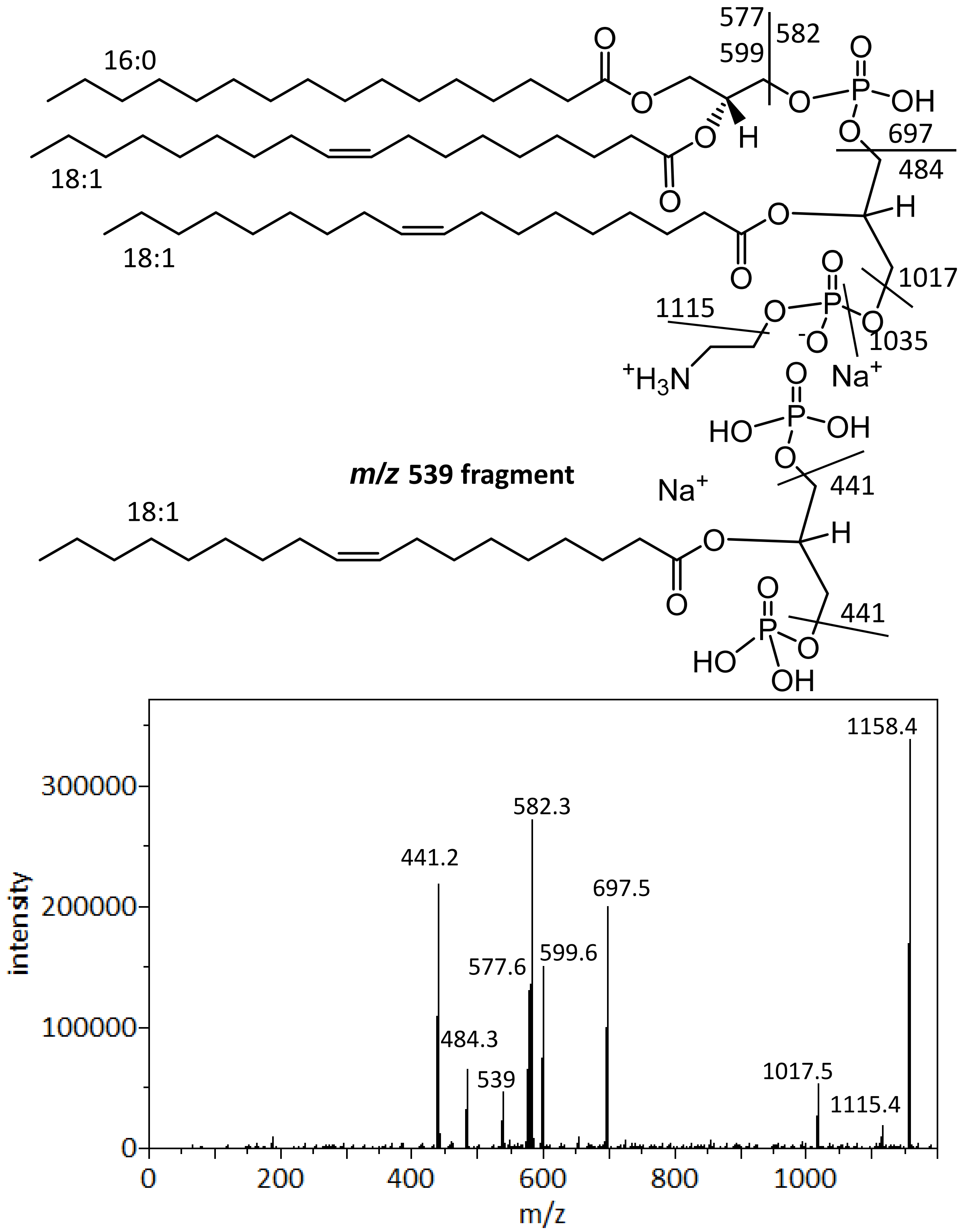

Figure S7C: Dissociation mechanism and MS/MS spectrum of *m/z* 1158 [PAGPE + Na]$^+$.

PAGPE was an unexpected finding in lipid extracts of *A. radioresistens* in the stationary phase. It appeared as peaks similar in size to LCL in the precursor scan for PE head group anion of *m/z* 140. The MS/MS spectrum of the most abundant *m/z* 1034 PAGPE anion is shown in Figure S7A. Signature fragments of PE at *m/z* 140 and 196 were observed as minor peaks (unlabelled in Figure S7A), corresponding to phosphoethanolamine and cyclic glycerophosphoethanolamine anions, respectively. There were two dominant fatty acid anions at *m/z* 255 and 281, respectively. Cyclic LPA anions with (16:0) and (18:1) fatty acyl were observed at *m/z* 391 and 417, respectively. Neutral loss of (18:1) FA or as ketene would produce fragments at *m/z* 852 and 870, respectively. Neutral loss of the (16:0) FA would produce the weaker *m/z* 878 anion. Importantly, the *m/z* 673 ion corresponds to PA anion with (16:0-18:1) fatty acyl composition, which was observed as the most common among PG, PE and CL. Neutral loss of a DG of this composition (594 amu) would produce the observed *m/z* 540 ion. Neutral loss of ethanolamine (61 amu) or cyclo-ethylamine (43 amu) would produce the pair of *m/z* 1073 and 1091 ions, while loss of linear or cyclic phosphoethanolamine (141 or 123 amu) would produce the pair of *m/z* 993 and 1011 ions. Observation of these four ions strongly suggest that the ethanolamine moiety is attached at the terminus, which would require that the third fatty acyl be attached to the central glycerol. Since all known glycerophospholipids are biosynthetically produced with phosphoester bonds to one of the two terminal hydroxyl groups in glycerol, we tentatively assign the acyl group to the central hydroxyl, while linking the phosphoethanolamine at the terminal hydroxyl. Therefore we name this lipid 1-phosphatidyl-2-acyl-glycero-3-phosphoethanolamine (PAGPE).

This assignment is consistent with the MS/MS spectrum of the corresponding protonated PAGPE ion at *m/z* 1136. As shown in Figure S7B, only two major fragments were observed. The *m/z* 577 fragment corresponds to the $[DG\text{-}OH]^+$ ion with a fatty acid composition of (16:0-18:1), while the *m/z* 462 ion likely corresponds to a neutral loss of PA (674 amu) which would produce the $[LPE\text{-}OH]^+$ ion with a fatty acyl composition of (18:1).

Sodiated PAGPE cation at *m/z* 1158 provided more fragmentation information, as shown in Figure S7C. The $[DG\text{-}OH]^+$ and $[DG\text{-}H2O\text{+}Na]^+$ twin peaks at 577 and 599 are consistent with the fatty acid composition of (16:0-18:1). So does the *m/z* 697 $[PA\text{+}Na]^+$ fragment. The *m/z* 582 fragment is likely due to neutral loss of a dehydrated DG with the assigned fatty acid composition, while the *m/z* 484 fragment is likely due to the loss of PA as assigned. The larger fragments of *m/z* 1115, 1035 (unlabelled minor peak) and 1017 likely corresponds to neutral loss of cyclic ethylamine (43 amu), cyclic phosphoethanolamine (123 amu) and phosphoethanolamine (141 amu), respectively. Detection of these three ions support the terminal assignment of the ethanolamine moiety. The *m/z* 539 fragment likely corresponds to the sodiated ion of the mid-section of the lipid molecule which is 2-acyl-glycerol-1,3-diphosphate. A further loss of a phosphoric acid (98 amu) would lead to the production of the *m/z* 441 fragment.

Fatty acid compositions of three observed PAGPE anions were assigned based on their MS/MS spectra, as listed in table S7. The two fatty acids attached to the first glycerol are listed with composition of the fatty acyl at the sn-1 position in the front. The fatty acyl positions were based on the observation that the fatty acid anion dissociated from the sn-2 position is more abundant than that from the sn-1 position. Fatty acyl composition of the second glycerol moiety is separated by a slash.

**Table S7. [PAGPE - H]$^-$ (*m/z*)**	**fatty acid composition**
1132	16:1-18:1 / 18:1
**1134**	**16:0-18:1 / 18:1**
1160	18:1-18:1 / 18:1

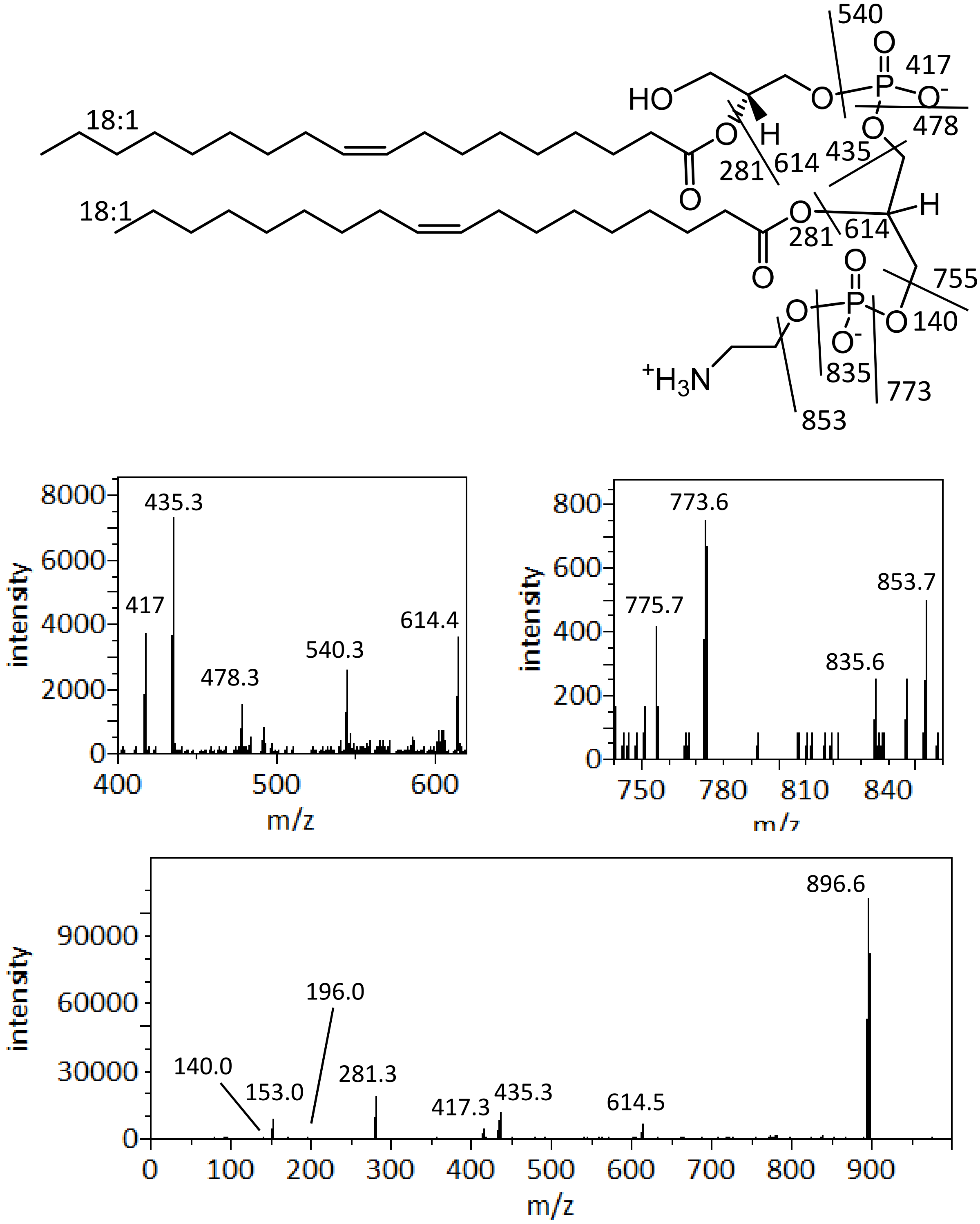

Figure S8: Dissociation mechanism and MS/MS spectrum of *m/z* 896 [LPAGPE - H]$^-$.

LPAGPE was only observed in cell in prolonged stationary phase. It was unexpectedly observed in the precursor scan for *m/z* 140 head group of PE. The MS/MS spectrum of the most abundant LPAGPE anion at *m/z* 896 is shown in Figure S8. There were only one dominant fatty acid anions at *m/z* 281, which is most likely located at the sn-2 position as observed in PG, PE and CL. The pair of LPA and cyclic LPA anions with (18:1) fatty acyl were observed at *m/z* 435 and 417, respectively. Neutral loss of one (18:1) FA (282 amu) would produce the fragment at *m/z* 614. The *m/z* 491 ion also corresponds to cyclic LPG ion with such a fatty acyl composition. Neutral loss of ethanolamine (61 amu) or cyclo-ethylamine (43 amu) would produce the weak pair of *m/z* 835 and 853 ions, while loss of linear or cyclic phosphoethanolamine (123 or 141 amu) would produce the pair of *m/z* 755 and 773 ions. Detection of these four ions strongly suggest that the ethanolamine moiety is attached at the terminus, implying that this ion is a lyso-form of PAGPE. Weaker ions at m/z 478 and 540 corresponds to neutral losses of a cyclic LPA (418 amu) and a monoacylglycerol (356 amu), respectively. This suggests that the first glycerol is likely mono-acylated and that the central glycerol must be acylated with the second fatty acyl group. Lack of a PA fragment ion at *m/z* 699 also supports this assignment.

Fatty acid compositions of two observed PAGPE anions at *m/z* 868 and 896 were assigned based on their MS/MS spectra, as listed in table S8. Fatty acyl composition on the two glycerol moiety is separated by a slash.

**Table S8. [LPAGPE - H]$^-$ (*m/z*)**	**fatty acid composition**
868	-16:1 / 18:1
896	-18:1 / 18:1

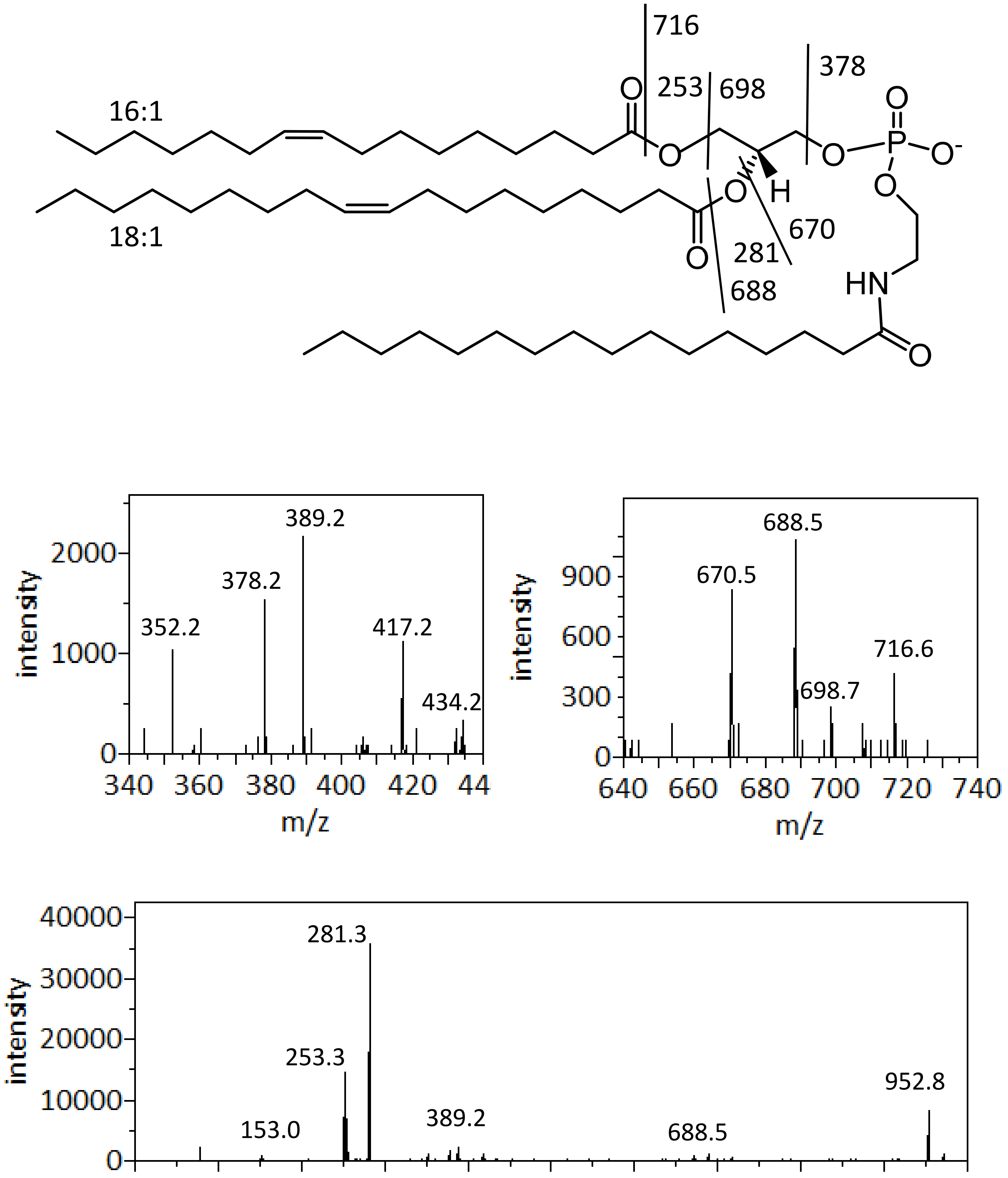

Figure S9: Dissociation mechanism and MS/MS spectrum of *m/z* 952 [NAPE-H]$^-$.

There were two anionic peak at *m/z* 952 and 954 in the negative mass spectra of lipids extracted from bacteria in the stationary phase. They also appeared in the fluorescent TLC band mainly composed of APG. We therefore acquired the MS/MS spectra of these two anions and assigned them as N-acyl-phosphatidylethanolamine (NAPE). As shown in Figure S9 and exactly as seen in the MS/MS spectrum of chemically synthesized NAPE reference, the N-acylated phosphoethanolamine head group was observed at *m/z* 378, corresponding to an N-fatty acyl of (16:0). Neutral loss of one fatty acid and the other as ketene from the phosphatidyl group would produce the other signature anion of NAPE at *m/z* 434. The two fatty acid anions at *m/z* 253 and 281 revealed the fatty acid compositions in the phosphatidyl group. The groups at the sn-1 and sn-2 positions were therefore assigned (16:1) and (18:1) fatty acyl, respectively. Two cyclic LPA anions at *m/z* 389 and 417 also supported this assignment. Neutral loss of a fatty acid or ketene at either of these two positions would produce the *m/z* 670 / 698 and 698 / 716 anionic pairs.

Fatty acid compositions of the two observed NAPE anions at *m/z* 952 and 954 were assigned based on their MS/MS spectra, as listed in table S9. Fatty acyl composition on the glycerol and the ethanolamine moieties is separated by a slash. The other even-numbered peaks nearby were of too low intensity to acquire meaningful MS/MS spectra.

**Table S9. $[NAPE - H]^-$ (*m/z*)**	**fatty acid composition**
952	16:1-18:1 / 16:0
954	16:0-18:1 / 16:0

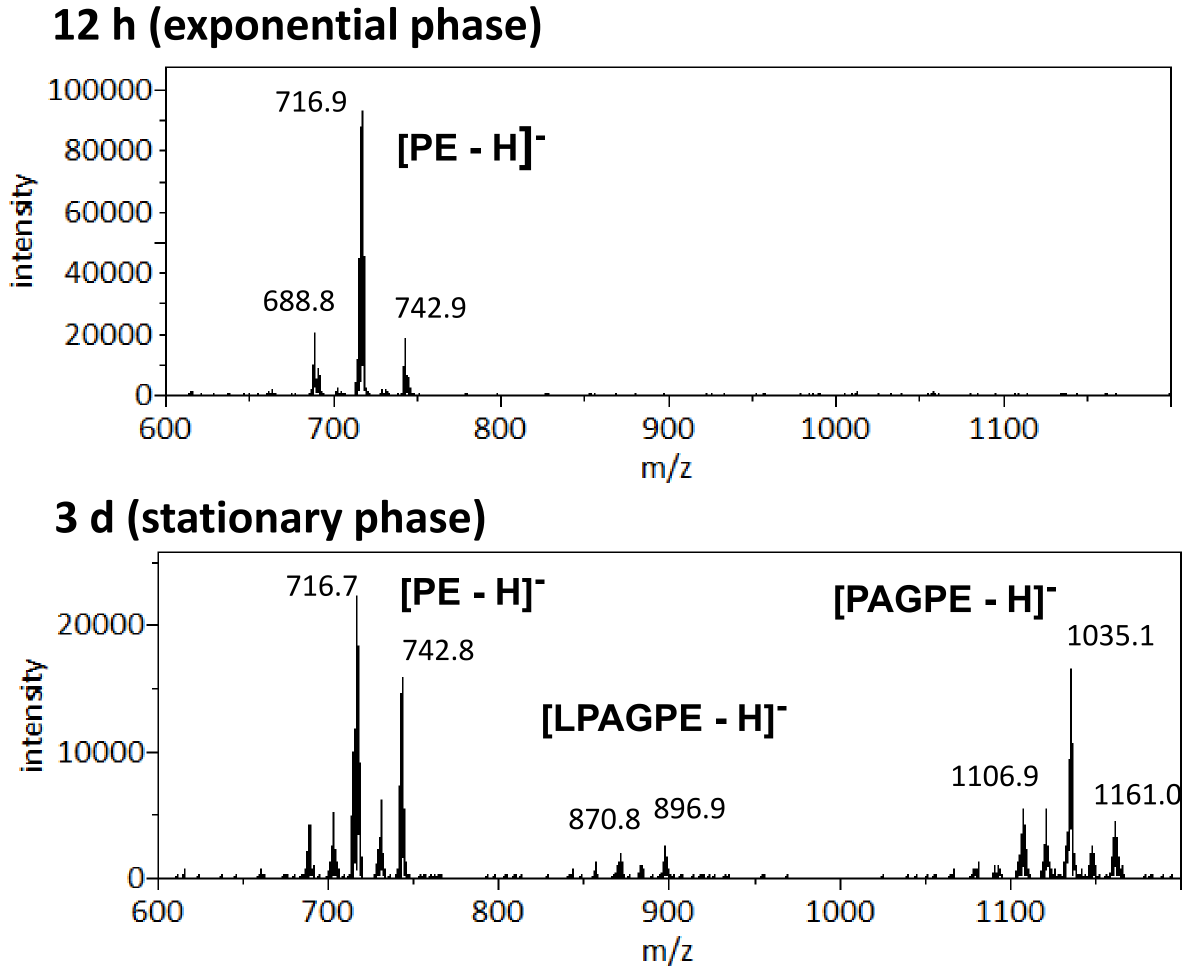

Figure S10: Precursor scan for *m/z* 140 head group fragment in PE and NAGPE.

A. calcoaceticus was obtained from the Culture Collection in the Department of Microbiology and Immunology. Two replicates of lipids were extracted from *A. calcoac*eticus during exponential phase 12 hours after inoculation, and stationary phase 3 days after inoculation. The MS precursor scans shown in Figure S10, as well as TLC and MS/MS experiments confirmed the detection of PAGPE and LPAGPE in lipids extracted during stationary phase. PAGPE and LPAGPE were not detected in *A. calcoaceticus* during exponential phase.

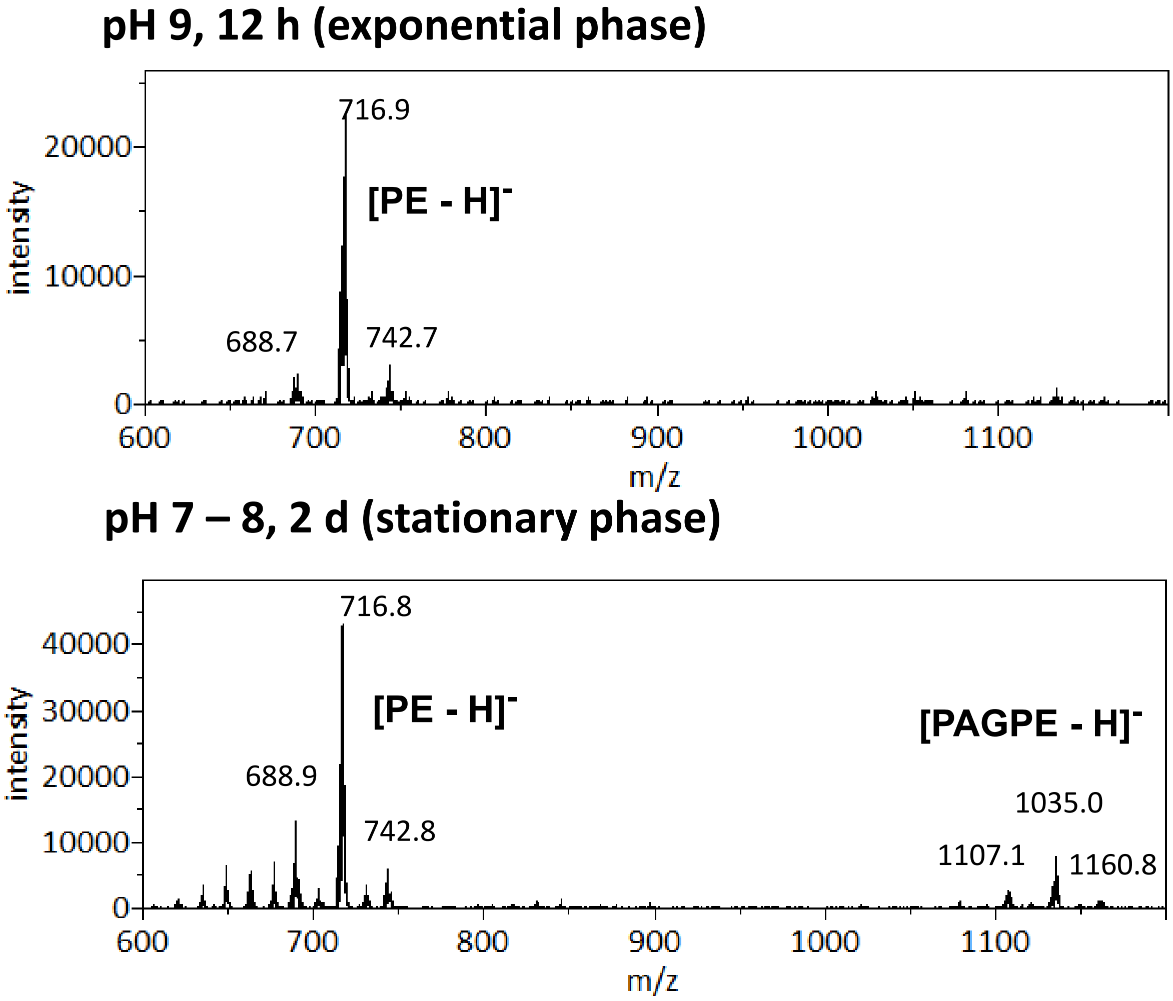

Figure S11: Precursor scan for *m/z* 140 head group fragment in PE and NAGPE.

A. Radioresistens was inoculated in LB media at pH 9.0 and pH 7.0 respectively and cultured at 37 °C. The pH 7 culture was monitored and supplemented with HCl to keep pH under 8.0. The MS precursor scans shown in Figure S11 confirmed the detection of PAGPE in lipids extracted during stationary phase at pH 7-8 but not during exponential phase at pH 9.0. This observation further supports the notion that PAGPE is only produced during stationary phase.

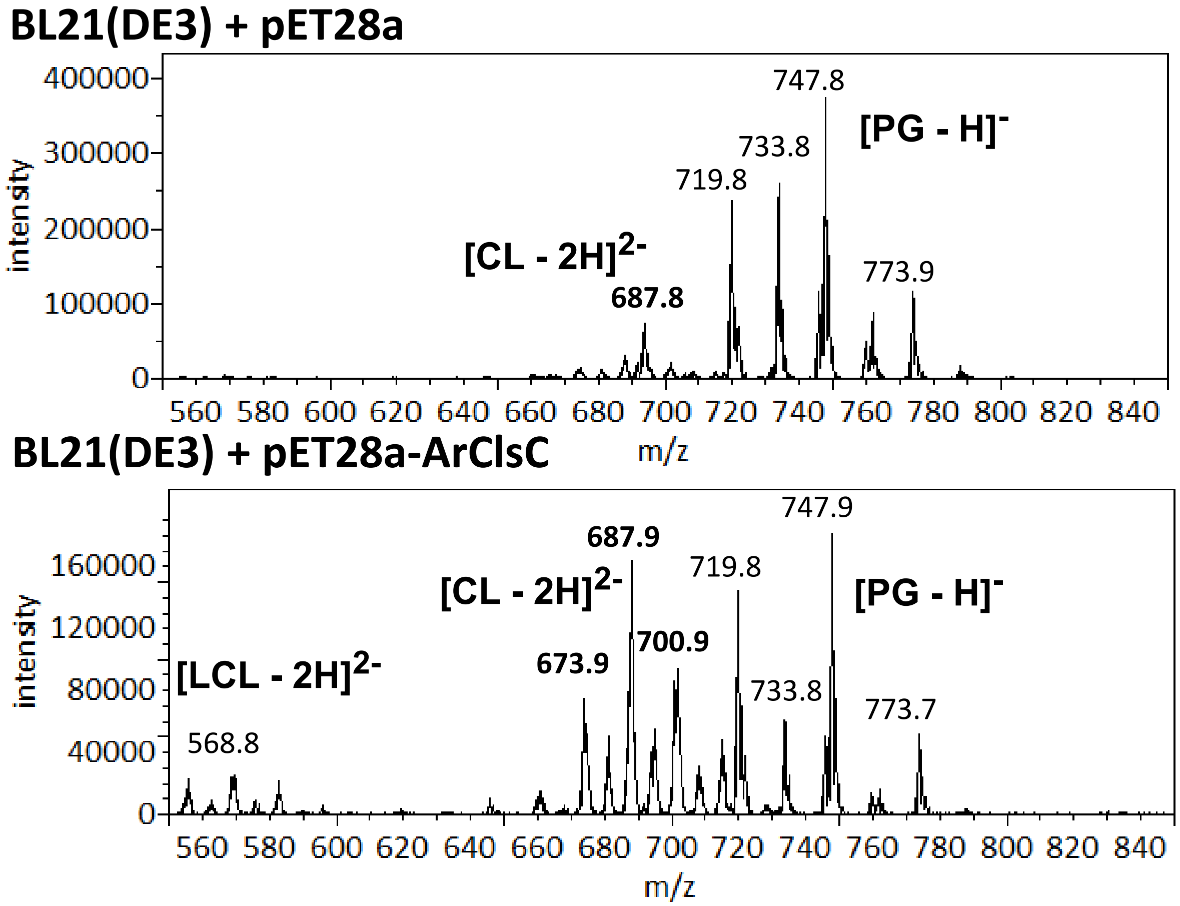

Figure S12: Precursor scan for *m/z* 153 head group fragment in PG, CL and LCL.

The open reading frame of the putative ClsC ortholog in *A. Radioresistens* (ArClsC) was synthesized and inserted into pET28a vector between Nco I and Xho I sites (Genscript). BL21(DE3) competent cells were transformed by heat shock at 42 °C for 45 s. Colonies on LB-Agar plates with 35 µg/ml kanamycin were inoculated in LB media. IPTG (1 mM) was added when cell density reached 0.5 during exponential phase. Cells were harvested by centrifugation after 1.5 h for lipid analysis.

The MS precursor scans shown in Figure S12 revealed a cluster (labels in bold) of CL double anions centered at *m/z* 687 and separated by 7, as well as a cluster of PG single anions center at *m/z* 747 and separated by 14 or 12. Lipids from BL21(DE3) cells transformed with pET-28a showed low abundance of CL, as we observed in untransformed BL21(DE3) cells during exponential phase. Over-expression of ArClsC noticeably increased concentration of both CL and LCL. TLC analysis of lipid composition was consistent with the MS analysis. These results support the notion ArClsC is a cardiolipin synthase.

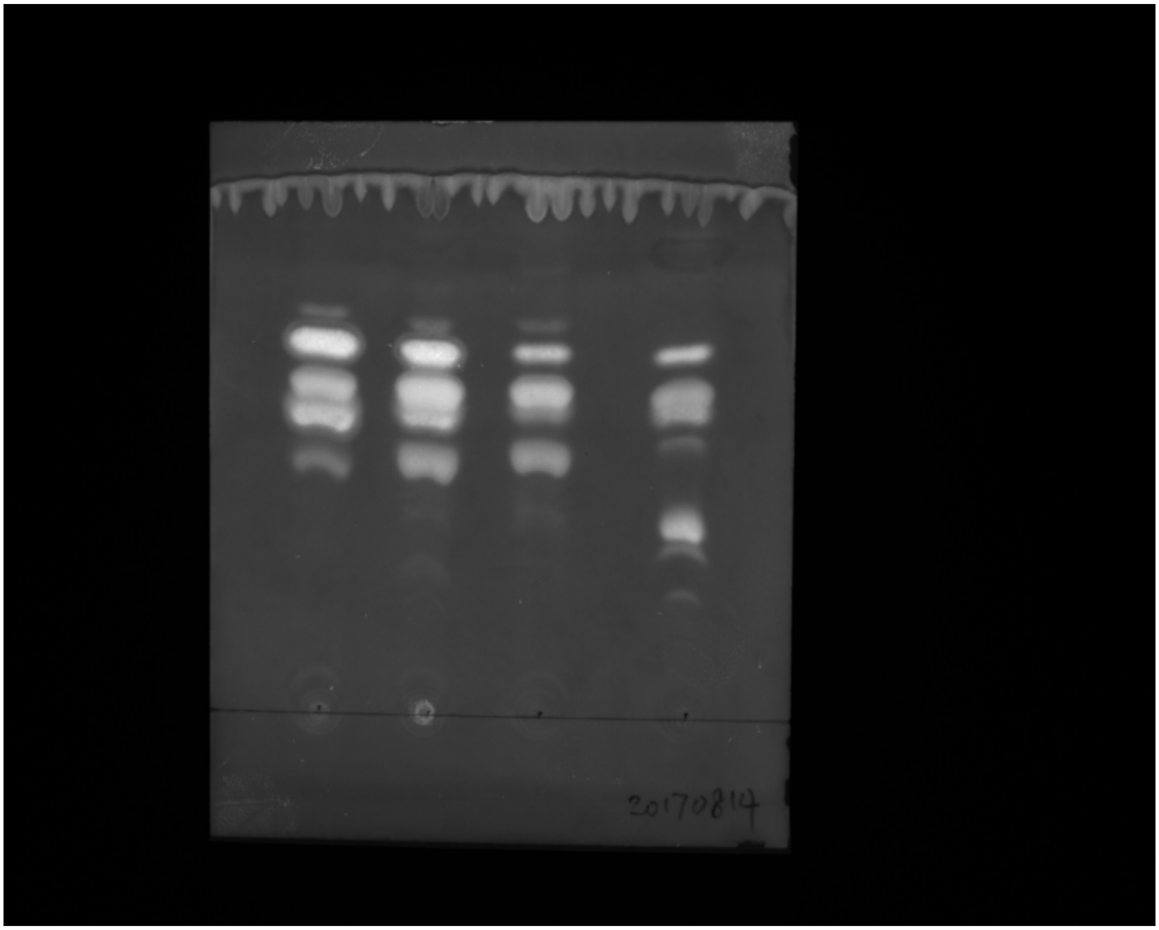

Original chromatogram of Figure 1A.

The chromatogram shown in Figure 1 was cropped from this original chromatogram. The TLC sheet was epi-illuminated by long wavelength UV. The fluorescence was filtered by a UV filter. No adjustment of brightness or contrast was done to the image. The default settings of brightness and contrast were used. The right-most lane in the original chromatogram corresponded to a sample from *Bacillus subtilis.* Therefore it was not shown in Figure 1.

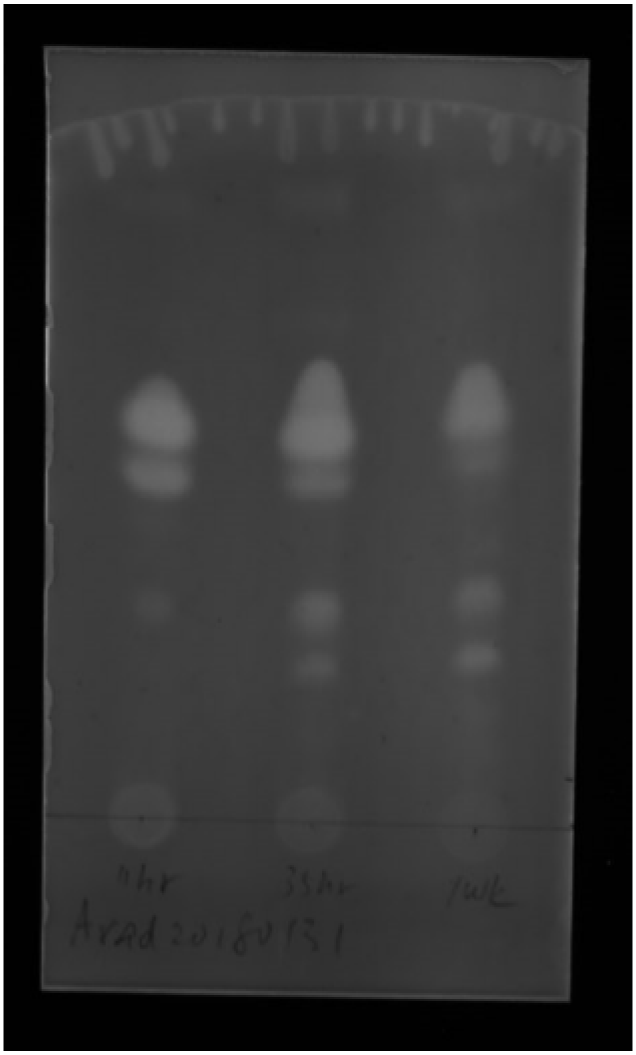

Original chromatogram of Figure 1B.

The chromatogram shown in Figure 1B was cropped from this original chromatogram. The TLC sheet was epi-illuminated by long wavelength UV. The fluorescence was filtered by a UV filter. No adjustment of brightness or contrast was done to the image. The default settings of brightness and contrast were used.

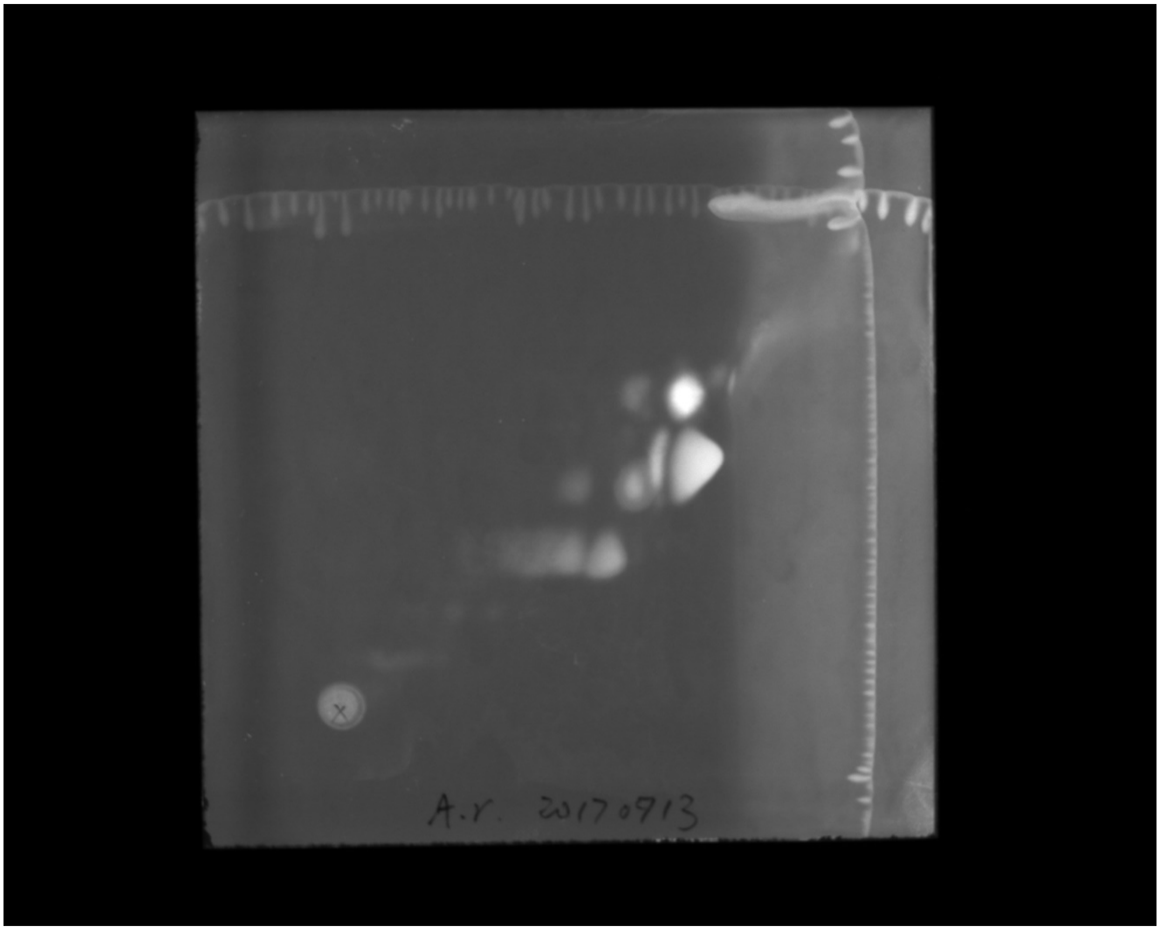

Original chromatogram of Figure 5.

The 2D-TLC chromatogram shown in Figure 5 was cropped from this original chromatogram. The TLC sheet was epi-illuminated by long wavelength UV. The fluorescence was filtered by a UV filter. No adjustment of brightness or contrast was done to the image. The default settings of brightness and contrast were used.
